# Development and validation of genome-wide polymorphic InDel marker set for harnessing the CC-genome wild rice species in the genus *Oryza*

**DOI:** 10.3389/fpls.2026.1733586

**Published:** 2026-01-28

**Authors:** Patricia Izabelle M. Lopez, Sherry Lou Hechanova, Charng-Pei Li, Sam Cohrs, Il-Ryong Choi, Pompe C. Sta. Cruz, Jose E. Hernandez, Tonette P. Laude, Sung-Ryul Kim

**Affiliations:** 1 Rice Breeding Innovation Department, International Rice Research Institute (IRRI), Los Baños, Philippines; 2 Institute of Crop Science (ICropS), College of Agriculture and Food Science (CAFS), University of the Philippines Los Baños (UPLB), Los Baños, Philippines; 3 Crop Science Division, Taiwan Agricultural Research Institute (TARI), Ministry of Agriculture, Taichung, Taiwan; 4 Department of Biology, Illinois Wesleyan University, Bloomington, IL, United States

**Keywords:** CC genome, InDel marker, *Oryza*, *Oryza eichingeri*, *Oryza officinalis*, *Oryza rhizomatis*, wild rice species, wild relative

## Abstract

Wild rice species serve as a valuable genetic reservoir for rice improvement, and the DNA segments containing useful genes/alleles can be transferred to elite rice varieties through crosses. However, DNA markers, which are essential tools for genetic analysis and molecular breeding, are not yet well-established for harnessing wild rice species. To enable an efficient utilization of the CC-genome wild rice species in the genus *Oryza*, we developed a genome-wide polymorphic InDel marker (≥20 bp) set comprising 182 markers through positional sequence alignments between *O. officinalis* (CC-genome) and rice cultivars (AA-genome). These markers were evenly distributed across the 12 chromosomes, with ~2Mb marker intervals. For validation of marker polymorphism, all the markers were tested by using PCR-agarose gel analysis with 12 accessions of CC-genome species (four accessions each from *O. eichingeri*, *O. officinalis*, and *O. rhizomatis*), two accessions of BBCC-genome species (*O. minuta*), and two cultivars, Nipponbare (*japonica*) and IR24 (*indica*). Out of 182 markers, 172 markers (94.5%) successfully amplified and exhibited polymorphism between rice and the CC-genome accessions, corresponding to an average marker interval of ~2.17Mb across the rice genome. Moreover, the marker set also showed high polymorphism (84.1-92.9%) when applied to BBCC-genome species. Based on the marker validation data, five markers were selected for species identification within the three CC-genome species. In addition, the polymorphic markers successfully detected wild rice introgressions from the wide hybridization progenies. The newly developed marker set will function as valuable genomic tools for harnessing CC-genome and CC-genome containing germplasm for rice improvement.

## 1 Introduction

Rice, one of the world’s staple food crops, is also one of the most economically and nutritionally important crops globally (Khush, 2005; Asma et al., 2023). Rice productivity is highly sensitive to both biotic and abiotic stresses, and if not mitigated, both yield and grain quality will significantly decline, and many farmers and consumers, especially in developing countries, will be affected. As a result, breeding institutions have prioritized developing high-yielding and stress-tolerant rice varieties. However, these efforts face the ongoing challenge of a narrowing genetic diversity of cultivated rice varieties due to domestication bottlenecks and selection (Zhu et al., 2007).

In contrast, wild rice species belonging to the genus *Oryza* are a valuable reservoir of key traits for sustainable varietal development (Brar and Khush, 1997). These species evolved naturally over millions of years in a broad range of normal to adverse environments without human intervention. Consequently, wild rice species possess genes/alleles that are associated with resistance to biotic stresses such as pests and diseases (Simon et al., 2023), as well as tolerance to abiotic stresses such as drought, submergence, flooding, salinity, and extreme temperatures (Zhang and Xie, 2014). Moreover, wild rice species also carry valuable agronomic traits which are not available from rice (*O. sativa*), such as extra-long-exserted stigmas for hybrid seed production (Prahalada et al., 2021), perennial growth habits of rice varieties for sustainable cropping systems (Zhang et al., 2023), and early morning flowering trait for heat avoidance during pollination (Uga et al., 2003).

There are 23 species comprising the genus *Oryza.* These species are grouped into 11 genome types (AA, BB, CC, EE, FF, GG, BBCC, CCDD, HHJJ, HHKK, and KKLL), including both diploids and allotetraploids (Vaughan et al., 2003). Among these, CC-genome species consisting of three species (*O. officinalis*, *O. rhizomatis*, and *O. eichingeri*) have been reported to have diverse valuable traits, including resistance to major insect pests like brown planthopper (BPH) (Jena and Khush, 1990). The *BPH14* gene, which confers resistance to BPH, was identified from *O. officinalis* and introgressed into cultivated rice (Du et al., 2009). In addition, a blast resistance gene, *Pi54*, was found in *O. rhizomatis* (Das et al., 2012). For agronomic traits, *O. officinalis* has also been associated with early morning spikelet opening, which is important in heat stress avoidance (Ishimaru et al., 2010). These traits highlight the potential of CC-genome species in improving resistance to biotic and abiotic stresses, as well as improving yield and grain quality in modern rice varieties.

Despite these advantages, integrating CC-genome traits into cultivated rice is a challenge due to reproductive barriers and a lack of genome-specific molecular tools tailored to these wild genomes (Brar and Khush, 1997). Molecular markers are useful tools in genetic analysis as well as modern plant breeding. They can facilitate trait mapping, gene discovery, genetic diversity analysis, and marker-assisted selection. In rice, simple sequence repeats (SSRs) and sequence-targeted sites (STSs) are the most widely used types of markers (McCouch et al., 2002). However, these markers are designed by using the sequences of rice (*O. sativa*: AA-genome), and when applied to wild rice species outside the AA-genome, they frequently fail to amplify clear bands, probably due to the large sequence difference at the primer annealing sites or show low levels of polymorphism. Consequently, using these markers to study or utilize wild rice species like those with the CC-genome is often inefficient and resource-intensive. In addition, the diagnostic marker sets which can discriminate species in the genus *Oryza* or species within the same genome types are not well established.

To address this challenges, genome-specific polymorphic markers through direct sequence comparisons between rice (*O. sativa*) and wild rice species should be developed. While previous studies successfully developed markers for AA-genome and BB-genome wild rice species (Hechanova et al., 2021; Malabanan-Bauan et al., 2023), the CC genome remains underrepresented in marker development efforts. This lack of a comprehensive and validated marker set slows down genetic exploration and breeding efforts involving CC-genome germplasm.

This study aimed to develop and validate a set of genome-wide insertion-deletion (InDels) markers showing polymorphism between cultivated rice and the CC-genome wild rice species. In total, 182 markers were designed through multiple sequence alignment between *O. officinalis* and five rice cultivars (*O. sativa*), while considering genome-wide distributions with ~2Mb marker intervals across all 12 rice chromosomes. Moreover, all the markers were evaluated by PCR-gel analysis with 14 accessions of CC-/BBCC-genome species and two rice cultivars (one *japonica* cultivar, Nipponbare and one *indica* cultivar, IR24). The newly developed marker set is intended to serve as a valuable resource to facilitate development of introgression populations, gene mapping, genetic analysis, and the introgression of key traits from the CC-genome into cultivated rice.

## 2 Materials and methods

### 2.1 Sequence preparation and sequence comparisons

The genome sequence data for the CC-genome species, the chromosome-level assembly sequence of *O. officinalis* (Shenton et al., 2020), was obtained from the DNA Databank of Japan (DDBJ: https://www.ddbj.nig.ac.jp/index-e.html). The genome sequences of four rice cultivars (IR8, IR64, Minghui 63, and Zhenshan 97) were downloaded from the NCBI nucleotide database. Inclusion of several cultivar sequences in the multiple sequence alignments was intended to select common InDel regions among cultivars rather than variety specific InDels. So that the potential polymorphic markers can show high polymorphism with many other rice cultivars. Approximately 20-kb bait sequences of Nipponbare were prepared from the RAP-DB (https://rapdb.dna.affrc.go.jp). To avoid long repeat regions such as transposable elements in bait sequence, the bait sequences were manually selected by using display option of ‘Repeat region’ under JBrowse, RAP-DB. The next bait sequences were manually moved with ~2Mb intervals along each chromosome, ensuring coverage across the genome. Orthologous regions were isolated from the bait sequence using the BLAST tool under NCBI Genome Workbench Software (Default parameters with Word Sizes: 70-120). Subsequently, multiple sequence alignment was performed using the web-based tool mVISTA (http://genome.lbl.gov/vista/), and polymorphic regions observed in the aligned sequences were highlighted and exported into a text file format using the BioEdit software (Hall, 1999).

### 2.2 Primer design

The multiple sequence alignment exported from the BioEdit software was opened using Microsoft Word. InDel regions containing gaps greater than 20 base pairs were manually screened among *O. officinalis* and the cultivars for clear separation in ~2% agarose gel. Forward and reverse primers were manually designed on the conserved regions flanking the polymorphic InDel sites to ensure proper primer annealing across all the species analyzed. Primer design followed a specific criterion: (1) GC content of approximately 50%; (2) annealing temperature around ~55°C; and (3) expected product sizes ranged from 100 to 500 bp. These parameters were laid out to standardize PCR conditions across all markers. Primer sequences were checked for redundancy using BLAST in RAP-DB, and only primers with unique hits were selected.

### 2.3 Plant materials

To validate the newly developed marker sets, PCR followed by agarose gel analyses was conducted using genomic DNA from wild and cultivated rice accessions. This panel included four accessions each of the CC-genome species, *O. eichingeri, O. officinalis, O. rhizomatis*, two accessions of BBCC-genome species *O. minuta*, and two Asian cultivated varieties: *O. sativa* spp. *indica* cv. IR24 and *O. sativa* spp. *japonica* cv, Nipponbare). All plant materials for the marker validation are listed in **Table 1**. Seeds of the wild rice species were obtained from the IRRI Genebank (https://gringlobal.irri.org/gringlobal/search), and wide-hybridization materials with CC-genome containing species were previously developed and maintained by the GIV laboratory at the IRRI. All the plant materials were grown in the glasshouse for wild rice species at the IRRI headquarters, Philippines.

**Table 1:** Summary of validation of the genome-wide 182 InDel markers.

Sample code	Species	Genome	Variety/Accession no.	Origin	No. of polymorphic markers (vs Nipponbare)	No. of polymorphic markers (vs IR24)	No. of no amplification marker
Nipponbare	*O. sativa*	AA	cv. Nipponbare	Japan	-	-	-
IR24	*O. sativa*	AA	cv. IR24	Philippines	-	-	-
Eichi_C01	*O. eichingeri*	CC	IRGC 101424	Uganda	165	165	2
Eichi_C02	*O. eichingeri*	CC	IRGC 99567	Tanzania	164	162	3
Eichi_C03	*O. eichingeri*	CC	IRGC 101424	Uganda	165	165	2
Eichi_C04	*O. eichingeri*	CC	IRGC 105412	Sri Lanka	164	162	3
Offi_C05	*O. officinalis*	CC	IRGC 100896	Thailand	169	168	7
Offi_C06	*O. officinalis*	CC	IRGC 80777	Philippines	172	170	1
Offi_C07	*O. officinalis*	CC	IRGC 104671	Malaysia	172	171	1
Offi_C08	*O. officinalis*	CC	IRGC 105966	Indonesia	172	171	2
Rhizo_C09	*O. rhizomatis*	CC	IRGC 105432	Sri Lanka	166	165	4
Rhizo_C10	*O. rhizomatis*	CC	IRGC 103410	Sri Lanka	165	164	18
Rhizo_C11	*O. rhizomatis*	CC	IRGC 103421	Sri Lanka	164	162	4
Rhizo_C12	*O. rhizomatis*	CC	IRGC 105949	Sri Lanka	172	170	1
Minu_C13	*O. minuta*	BBCC	IRGC 101141	Philippines	169	167	4
Minu_C14	*O. minuta*	BBCC	IRGC 93257	Philippines	155	153	13

### 2.4 Leaf sampling and DNA extraction

Leaves approximately 2–4 cm were collected from the plant materials. Each leaf was directly placed in a 2-mL microcentrifuge tube containing two stainless steel balls inside. DNA extraction was carried out using a modified simple protocol based on Kim et al. (2016). A volume of 400 μL of TPE buffer (100 mM Tris-HCl, pH 9.5, 1 M KCl, 10 mM EDTA, pH 8.0) was added to each tube. Samples were then homogenized using a 2010 Geno/Grinder^®^ at 1000 RPM for 7 min. Following homogenization, the samples were incubated in a hot water bath at 65°C for 60 min. After incubation, tubes were centrifuged at 13000 × g for 15 min. The supernatant, containing the extracted genomic DNA of the samples, was transferred to 1.5-mL microcentrifuge tubes and stored at 4°C. For PCR analysis, each DNA sample was diluted by transferring 30 μL of crude DNA extracts into a 96-well plate containing 120 μL of double-distilled water and used as template DNA.

### 2.5 PCR and gel analysis

For PCR analysis, a 15 μL PCR mixture was prepared containing the following components: 10x PCR buffer, 200 μM of deoxyribonucleotide triphosphate (dNTP), 0.50 μM each of the forward and reverse primers, 1 unit of *Taq* DNA polymerase (BioFact, https://www.bio-ft.com/eng/index.html), 2 μL of the DNA and double distilled water to reach the final volume. Approximately 8 μL mineral oil was added to the reaction to avoid evaporation during thermal cycling. PCR was performed using thermal cyclers (Applied Biosystems VeritiTM 96- or 384-Well Fast Thermal Cycler, http://www.thermofisher.com) with reactions placed in either 96- or 384-well plates. The thermal cycling profile used for marker validation was as follows: an initial denaturation at 94°C for 2.5 min; 35 cycles of 95°C for 25 s, 55°C for 25 s, 72°C for 50 s; followed by a final extension at 72°C for 5 min. PCR products were separated on ~2% agarose gels.

### 2.6 Graphical mapping of markers in the rice genome

The physical positions of the developed markers were visualized on the 12 rice chromosomes using the web-based tool PhenoGram (http://visualization.ritchielab.psu.edu).

## 3 Results

### 3.1 Development of a genome-wide InDel marker set for the CC-genome *Oryza* species

We used a previously established strategy of multiple sequence alignment and primer design (Hechanova et al., 2021; Malabanan-Bauan et al., 2023) to develop evenly distributed genome-wide InDel markers that can distinguish alleles between cultivated rice (*O. sativa*) and CC-genome wild rice species. Specifically, bait sequences were obtained from the rice reference genome available at RAP-DB and used to retrieve orthologous genomic regions from the publicly available CC-genome species (*O. officinalis*) and cultivated rice varieties (AA-genome).

In certain instances, orthologous regions were not detected. This indicates possible absence or high divergence of those regions in wild rice species or some cultivars. Only orthologous sequences shared between *O. officinalis* and the two cultivars were retained for multiple sequence alignment. Alignments were generated using the mVISTA web-based tool, and sequence variations such as SNPs and InDels were identified using BioEdit. For clear separation in agarose gel, InDel regions showing at least a 20-bp gap between *O. officinalis* and the cultivars were selected for marker development.

Marker development was initiated from the tip of the short arm of each chromosome and was subsequently moved down in ~2Mb intervals to the next markers for uniform genomic coverage. A total of 182 markers were designed and distributed across the 12 rice chromosomes. Expected PCR amplicon sizes for each marker were calculated based on the alignment results for the two rice cultivars and *O. officinalis* (**Supplementary Table S1**).

**Figure 1:**
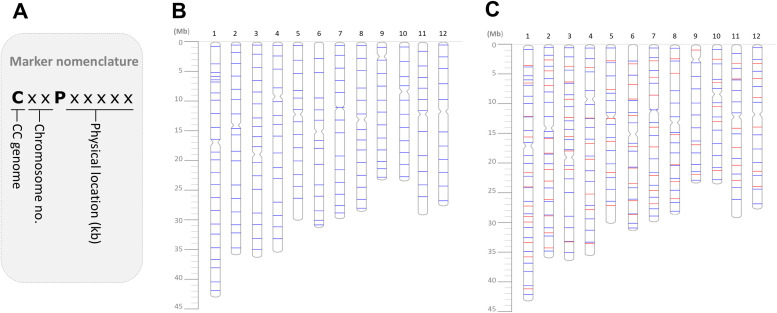
Nomenclature and physical locations of the CC-genome markers across the 12 chromosomes. **(A)** Marker nomenclature. **(B)** Distributions of the 172 polymorphic markers validated in this study. **(C)** Distributions of the 172 polymorphic markers (blue bars) together with the previously reported 87 polymorphic markers (Hechanova et al., 2018) (red bars).

Each marker was named using the format CxxPxxxxx, where “Cxx” denotes the genome type and chromosome number, and “Pxxxxx” represents the physical location (in kilobases) on the chromosome of the rice reference genome, Nipponbare (**Figure 1A**). This naming convention facilitates easy identification of both chromosomal and physical locations of each marker.

### 3.2 Marker validation with CC-genome *Oryza* species

To validate the developed markers, all 182 markers underwent PCR amplification followed by agarose gel analysis using genomic DNA from a *japonica* cultivar (Nipponbare)*, indica* cultivar (IR24), four accessions each of CC-genome wild rice species *O. eichingeri, O. officinalis, O. rhizomatis*, and two accessions of BBCC-genome wild rice species *O. minuta.* Out of the 182 markers, 172 markers (94.5%) successfully amplified and showed polymorphism between rice and one accession of CC-genome germplasm, with an estimated average marker interval of ~2.17Mb across the rice reference genome (IRGSP-1.0), while 10 markers showed no/weak amplification or produced unexpected multiple bands. All the gel images of the 182 markers and marker information are shown in the **Supplementary Figure S1** and **Supplementary Table S1**, respectively.

Among the 12 accessions under CC-genome, three *O. officinalis* accessions (Offi_C06, Offi_C07, Offi_C08) and one *O. rhizomatis* accession exhibited the highest number of polymorphic markers (172) when compared against Nipponbare (**Table 1**). Similarly, two *O. officinalis* accessions (Offi_C07, Offi_C08) displayed the highest number of polymorphic markers (171) against IR24. In contrast, lowest number of polymorphic markers for CC-genome were observed in two *O. eichingeri* accessions (Eichi_C02, Eichi_C04) and one *O. rhizomatis* accession (Rhizo_C11) with 164 and 162 polymoprhic markers when compared to Nipponbare and IR24, respectively. The CC-genome markers were also tested with two accessions of the BBCC-genome species, *O. minuta*. The results showed high polymorphisms (153 to 159 polymorphic markers) between cultivars and *O. minuta* (**Table 1**). The 172 markers exhibiting polymorphisms were evenly distributed across all 12 chromosomes (**Figure 1B**). In addition, 87 previously reported polymorphic markers developed and validated by Hechanova et al. (2018) were mapped alongside the newly designed polymorphic markers (**Figure 1C**), providing a comprehensive marker resource with an estimated average marker interval of ~1.44Mb for genetic studies in CC- and BBCC-genome wild rice species.

### 3.3 Selection of the species-specific markers within the CC-genome species

**Figure 2:**
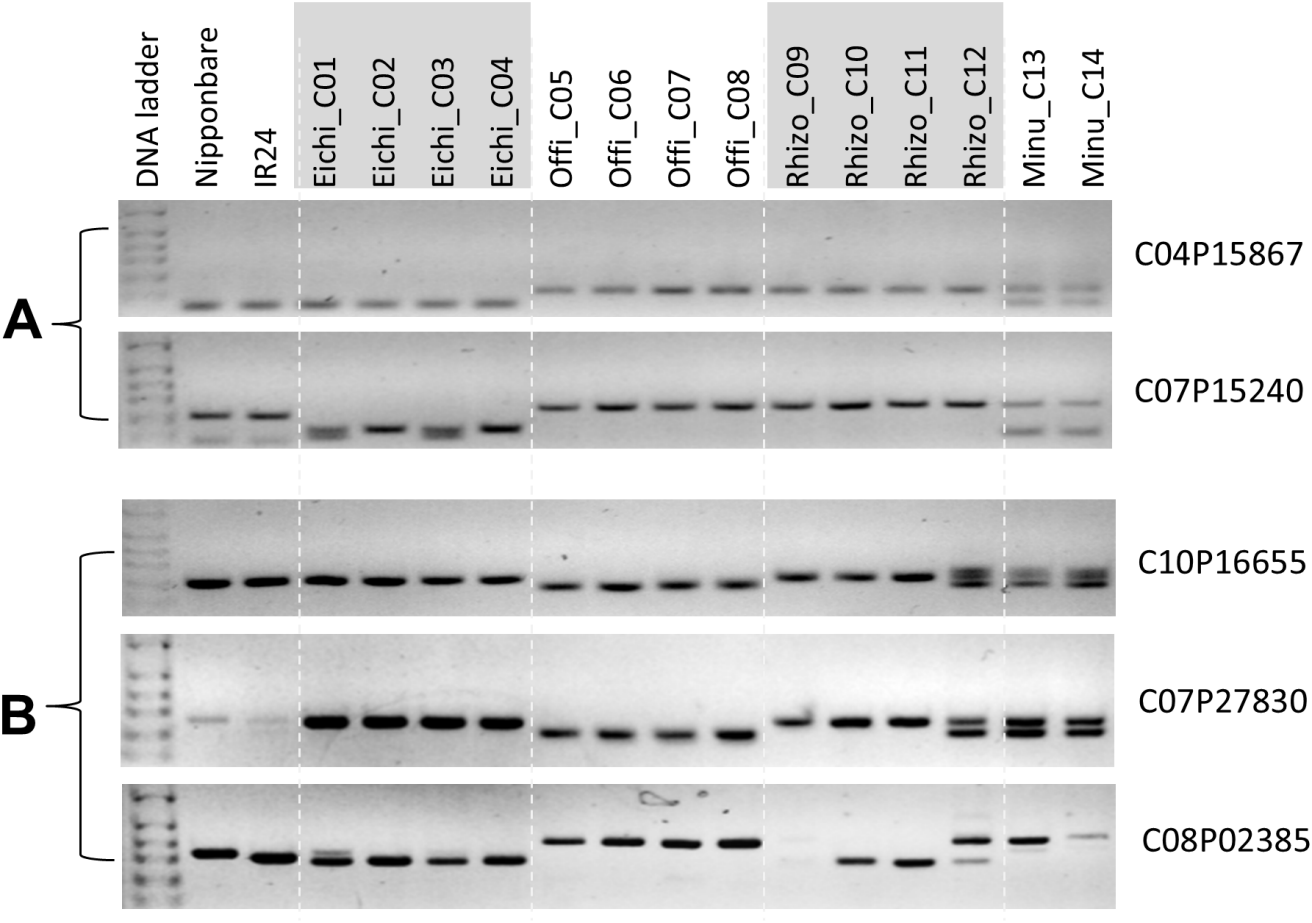
Species-specific markers that can discriminate the three CC-genome species (*O. eichingeri*, *O. officinalis*, and *O. rhizomatis*). **(A)***O. eichingeri* specific markers (C04P15867, C07P15240). **(B)***O. officinalis* specific markers (C10P16655, C07P27830, C08P02385).

By using the marker validation data, we isolated the species-specific markers that can discriminate the three CC-genome species. Two markers (C04P15867 and C07P15240) showed specific band patterns for *O. eichingeri* accessions, and three markers (C10P16655, C07P27830, and C08P02385) were specific for *O. officinalis* accessions (**Figure 2**). However, we were not able to find *O. rhizomatis* specific markers. These five markers would be useful to determine the species within the CC-genome species.

### 3.4 Application of the newly developed CC-genome markers in the hybrids with the CC-genome-containing wild species

**Figure 3:**
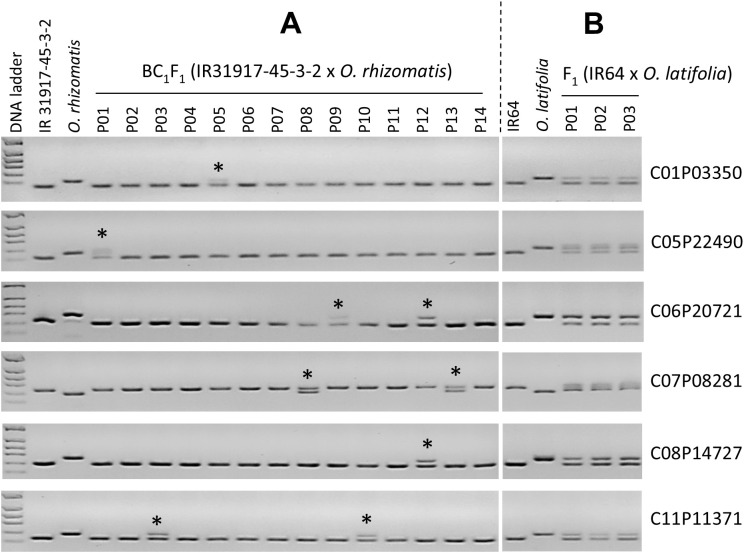
Applications of the newly developed CC-genome markers. **(A)** Detection of introgression of *O. rhizomatis* from the BC_1_F_1_ plants derived from the cross between the elite breeding line (IR31917-45-3-2) and *O. rhizomatis* (IRGC 105432). Asterisks mean *O. rhizomatis* introgression. **(B)** Hybridity tests from the F_1_s between IR64 and *O. latifolia* (CCDD-genome type, IRGC 101405).

We tested the newly developed CC-genome markers with the CC-genome-containing introgression lines in rice. Six markers showing polymorphism between rice and CC-genome species were randomly selected and applied for the detection of *O. rhizomatis* introgressions from 14 individual BC_1_F_1_ plants derived from the elite rice breeding line (IR31917-45-3-2) x *O. rhizomatis* cross. As expected, all six markers could detect introgressions of different chromosomes (**Figure 3A**). In addition, the same six markers were used to check true F_1_ hybridity from the cross between IR64 and *O. latifolia* (CCDD-genome) which contains CC genomes. Interestingly, all six markers also showed polymorphism between IR64 and CCDD-genome species (*O. latifolia*) and the F_1_ plants possessed both parental bands (**Figure 3B**), suggesting all three F_1_ plants were true hybrids. These results suggested that the newly developed CC-genome markers might be useful to harness the CC-genome species and also the CC-genome containing species such as BBCC and CCDD-genome species.

## 4 Discussion

Wild rice species exhibit extensive genetic diversity, having evolved and adapted to a broad range of environmental conditions. These wild relatives of rice serve as a valuable reservoir of genes related to resistance to various biotic and abiotic stresses, as well as traits related to yield improvement (Brar and Khush, 1997; Kumar et al., 2024). To effectively develop mapping populations, map the genes, dissect traits, and introgress useful genes/alleles to cultivated backgrounds, utilizing genome-specific molecular markers would be advantageous. For rice, the commonly used molecular markers are STS and SSR markers, which are developed based on the sequences of *O. sativa*. Although these markers are effective for discriminating allele variations for rice cultivars, application to more genetically distant wild rice relatives often requires time-consuming screening with several hundred markers, and PCR amplification used to show weak bands or absence in wild rice species, especially those outside AA-genome species, resulted in obtaining poor polymorphisms. Furthermore, it frequently showed uneven marker distributions across the genome.

To address this limitation, a genome-wide InDel marker set showing high polymorphism, specifically to CC-genome wild rice species was developed. Using a multiple sequence alignment and primer design strategy previously applied to the AA-genome (Hechanova et al., 2021) and BB-genome (Malabanan-Bauan et al., 2023), a total of 182 markers were initially designed. Among these, 172 (94.5%) successfully showed polymorphism across a panel of CC-genome accessions. High levels of polymorphism were obtained between rice cultivars and CC-genome containing species because all the markers were designed based on the direct sequence comparisons between rice and CC-genome species (*O. officinalis*), not like just extractions of simple sequence repeat (SSR) regions. Notably, three *O. officinalis* accessions and one *O. rhizomatis* accession exhibited the highest polymorphism rates, with up to 172 markers differentiating them from *O. sativa*. The high amplification and polymorphism rates across the panel emphasize the utility of these markers for genetic analysis of CC-genome species.

In addition, the developed markers were also effective in the two accessions of the BBCC-genome species *O. minuta*, with polymorphism levels ranging from 153 to 169 markers (84.1-92.9%). High frequency of polymorphic markers might be achieved by presence of the CC genome in the *O. minuta* but the sequence divergence during the independent evolution of CC and BBCC genome species might cause slightly reduced polymorphism compared to CC-genome species. This result aligns with the previous findings showing reduced amplification and polymorphism when markers designed for one genome type are applied to more distant relatives (Chin et al., 2017; Malabanan-Bauan et al., 2023). Thus, the development of the genome-type-specific marker set would provide high polymorphism.

All polymorphic markers were evenly distributed across the 12 chromosomes, providing genome-wide coverage and facilitating their use in applications such as the development of mapping populations, QTL mapping, and diversity studies. All markers were designed to yield clear banding patterns on standard agarose gels (~2.0% agarose), which offers a cost-effective and user-friendly alternative to more technically complex techniques such as PAGE or DNA fragment analysis. In addition to the newly developed 172 polymorphic markers, we mapped 87 previously reported markers (Hechanova et al., 2018) across the 12 rice chromosomes. These 259 potential polymorphic markers will provide higher marker density (~1.44Mb marker intervals) and also high chances of polymorphism with diverse CC-genome containing accessions and rice varieties for genetic analysis and utilization of CC-genome-containing germplasm.

The Linnaean classification system is highly dependent on morphological characteristics, and sometimes it causes conflict among taxonomists. Three CC-genome species are phenotypically similar to each other (**Supplementary Figure S2**). Through marker validations with 182 markers, we identified two (C04P15867 and C07P15240) and three markers (C10P16655, C07P27830, and C08P02385) that showed specific band patterns for *O. eichingeri* accessions and *O. officinalis* accessions, respectively. These five markers can be potentially used for species identification within CC-genome species.

Overall, the development of the CC-genome-specific InDel markers offers a valuable resource for genetic studies and breeding efforts involving wild rice species. The high amplification efficiency, even distribution across the genome, and robust polymorphism make them useful for identifying important genes/alleles from the CC-genome species. Furthermore, utilization in CC-genome containing species (BBCC- and CCDD-genome species) highlights the versatility of the markers. These markers can significantly reduce the time, cost, and labor involved in marker screening and can aid in the efficient use of the genetic diversity from wild rice species.

## Data Availability

The datasets presented in this study can be found in online repositories. The names of the repository/repositories and accession number(s) can be found in the article/**Supplementary Material**.
