# Supplementary material for: Development and validation of genome-wide polymorphic InDel marker set for harnessing the CC-genome wild rice species in the genus Oryza
Source: Front Plant Sci. 2026 Jan 28;17:1733586. doi: 10.3389/fpls.2026.1733586 (PMC12891111; doi:10.3389/fpls.2026.1733586)
Supplement: Supplementary file 2 [file DataSheet1.pdf]

Supplementary Figure S1. Agarose gel images of the CC-genome InDel markers. All the 182 markers were analyzed by PCR-gel analysis with 12 accessions of CC-genome species (four accessions of *O. eichingeri*, *O. officinalis*, and *O. rhizomatis*), two accessions of BBCC-genome species (*O. minuta*), and two cultivars (Nipponbare and IR24). Sample codes, DNA ladders, and gel images of all the markers are presented here.

[Sample code]

Nipponbare
IR24
Eichi_C01
Eichi_C02
Eichi_C03
Eichi_C04
Offi_C05
Offi_C06
Offi_C07
Offi_C08
Rhizo_C09
Rhizo_C10
Rhizo_C11
Rhizo_C12
Minu_C13
Minu_C14

[DNA ladder]

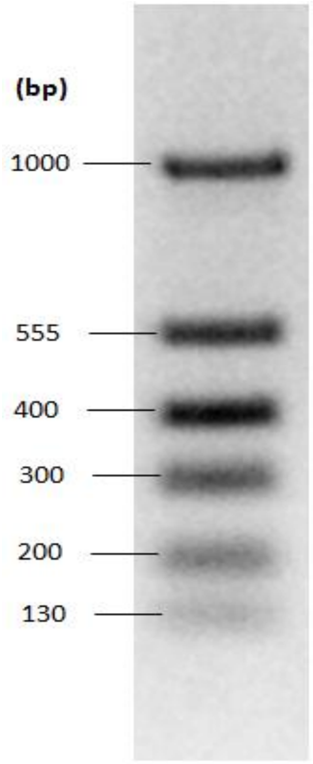

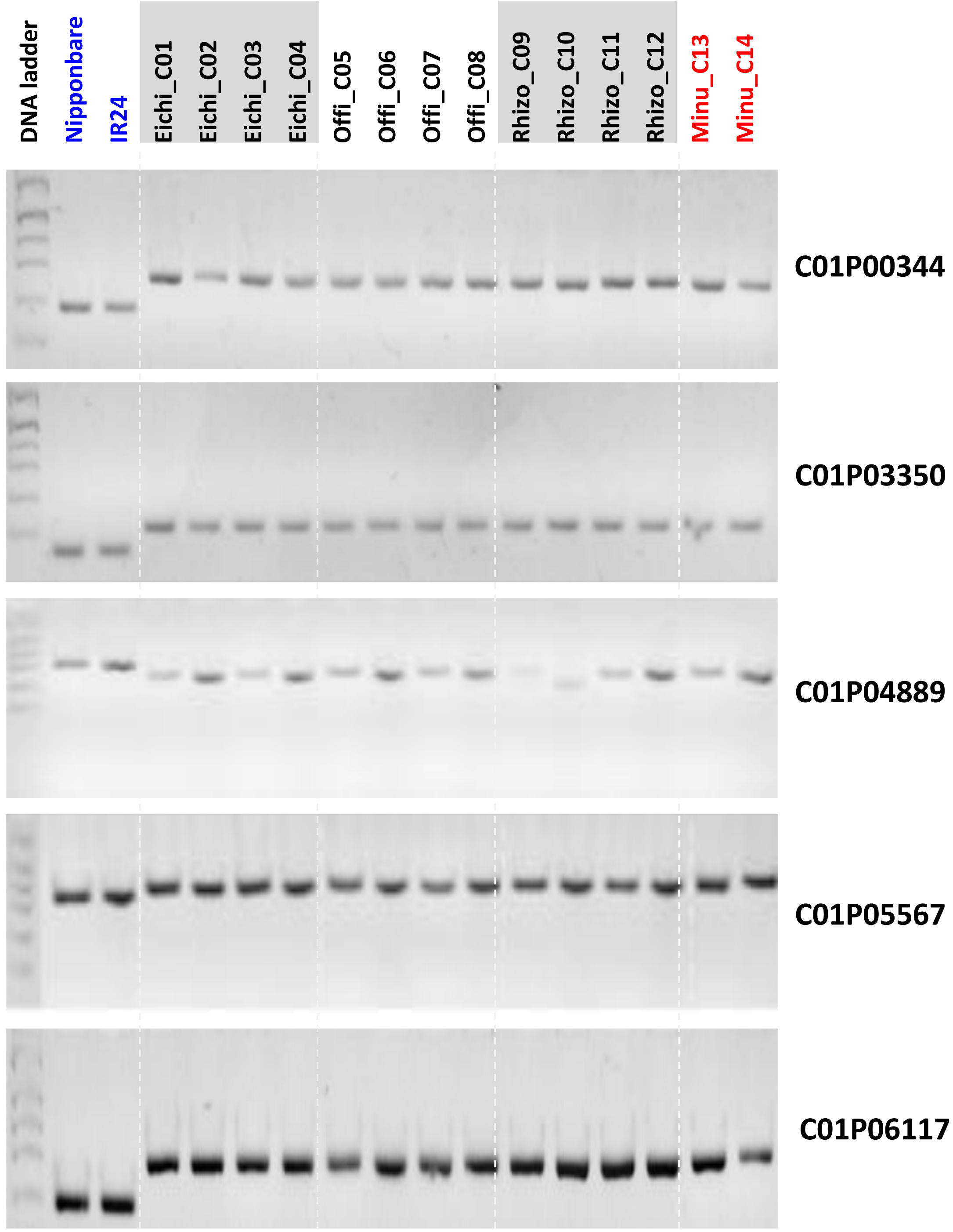
DNA ladder
Nipponbare
IR24
Eichi_C01
Eichi_C02
Eichi_C03
Eichi_C04
Offi_C05
Offi_C06
Offi_C07
Offi_C08
Rhizo_C09
Rhizo_C10
Rhizo_C11
Rhizo_C12
Minu_C13
Minu_C14
C01P00344
C01P03350
C01P04889
C01P05567
C01P06117

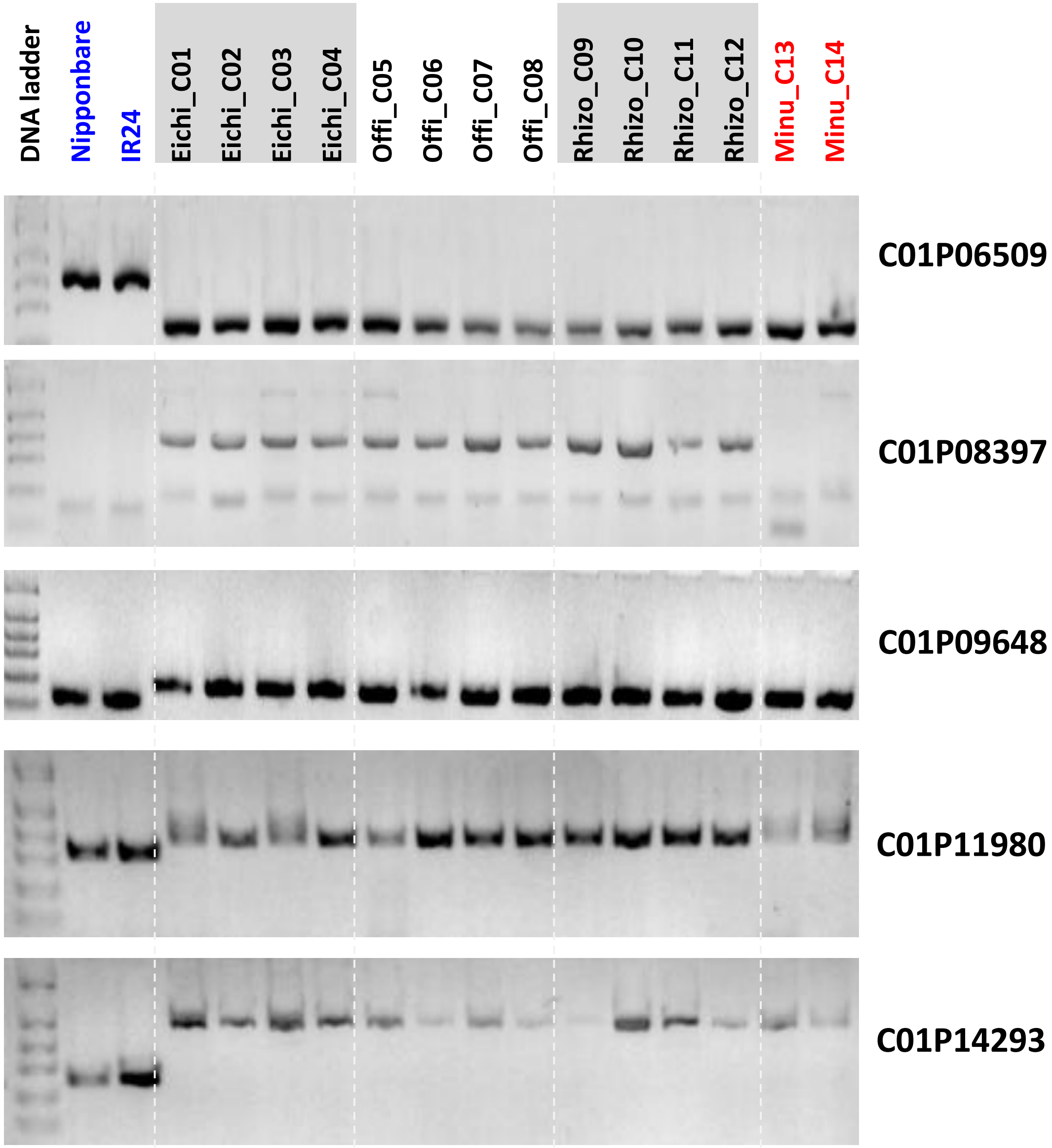
DNA ladder
Nipponbare
IR24
Eichi_C01
Eichi_C02
Eichi_C03
Eichi_C04
Offi_C05
Offi_C06
Offi_C07
Offi_C08
Rhizo_C09
Rhizo_C10
Rhizo_C11
Rhizo_C12
Minu_C13
Minu_C14
C01P06509
C01P08397
C01P09648
C01P11980
C01P14293

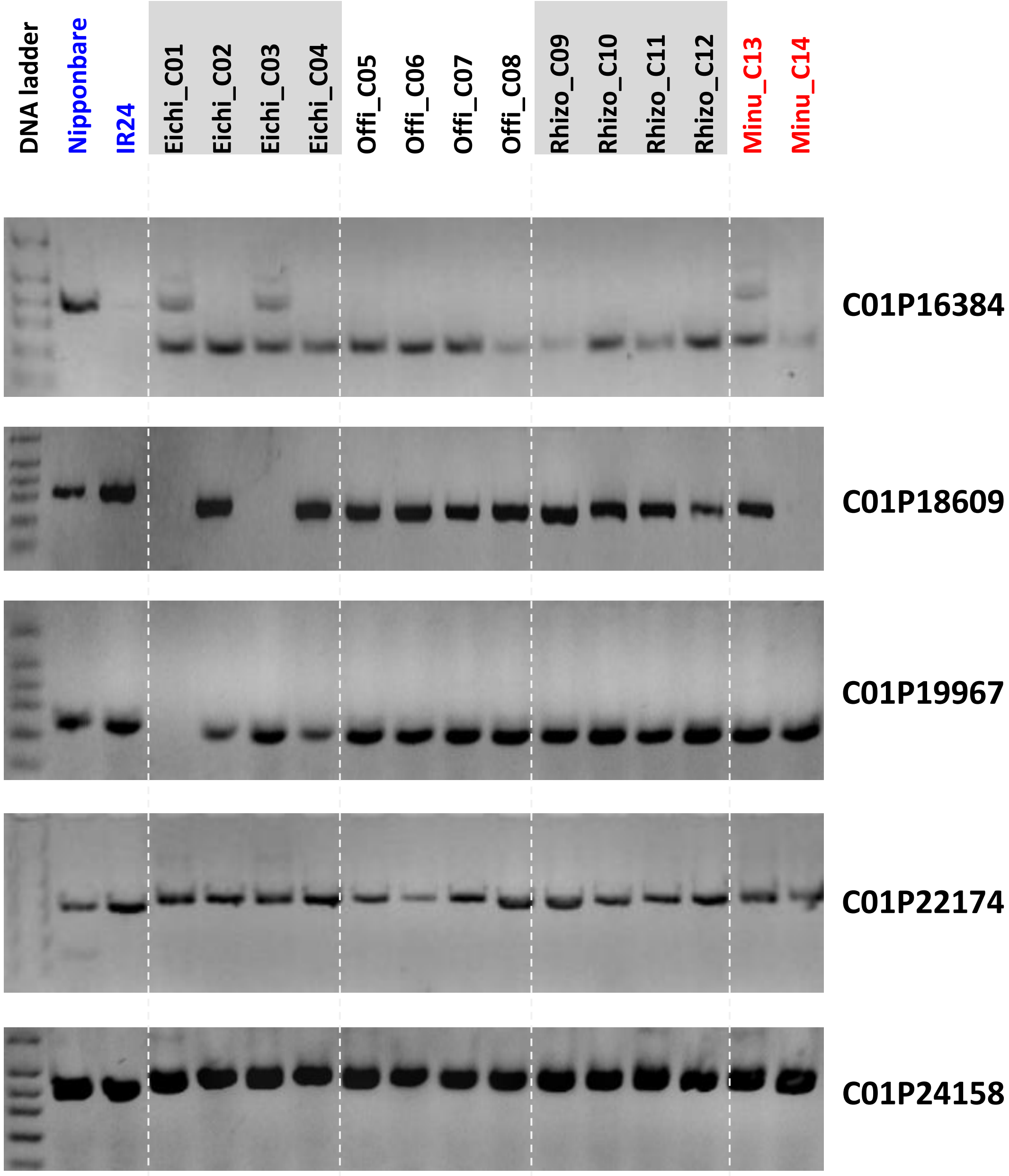
DNA ladder
Nipponbare
IR24
Eichi_C01
Eichi_C02
Eichi_C03
Eichi_C04
Offi_C05
Offi_C06
Offi_C07
Offi_C08
Rhizo_C09
Rhizo_C10
Rhizo_C11
Rhizo_C12
Minu_C13
Minu_C14
C01P16384
C01P18609
C01P19967
C01P22174
C01P24158

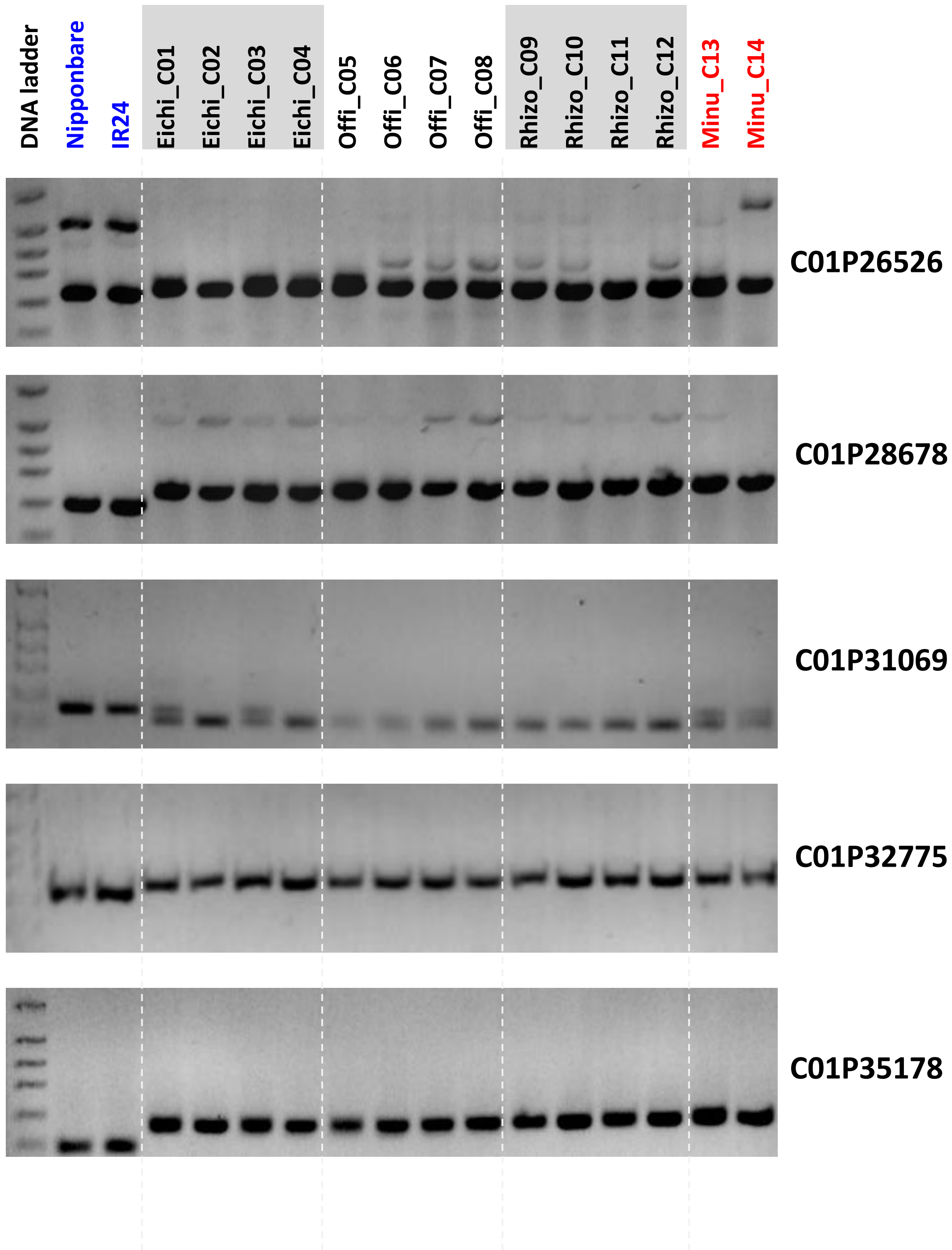

DNA ladder
Nipponbare
IR24
Eichi_C01
Eichi_C02
Eichi_C03
Eichi_C04
Offi_C05
Offi_C06
Offi_C07
Offi_C08
Rhizo_C09
Rhizo_C10
Rhizo_C11
Rhizo_C12
Minu_C13
Minu_C14
C01P26526
C01P28678
C01P31069
C01P32775
C01P35178

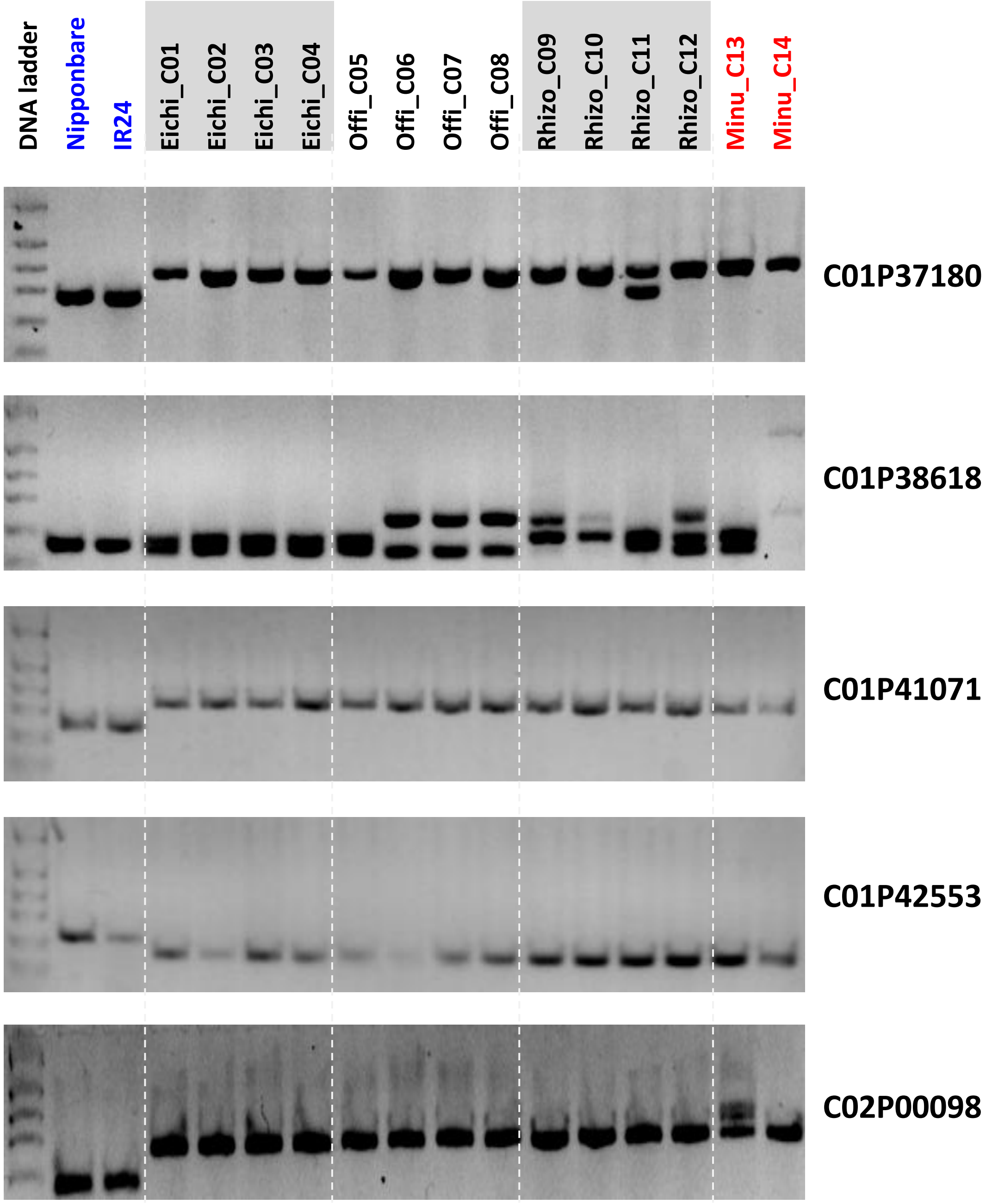

DNA ladder
Nipponbare
IR24
Eichi_C01
Eichi_C02
Eichi_C03
Eichi_C04
Offi_C05
Offi_C06
Offi_C07
Offi_C08
Rhizo_C09
Rhizo_C10
Rhizo_C11
Rhizo_C12
Minu_C13
Minu_C14
C01P37180
C01P38618
C01P41071
C01P42553
C02P00098

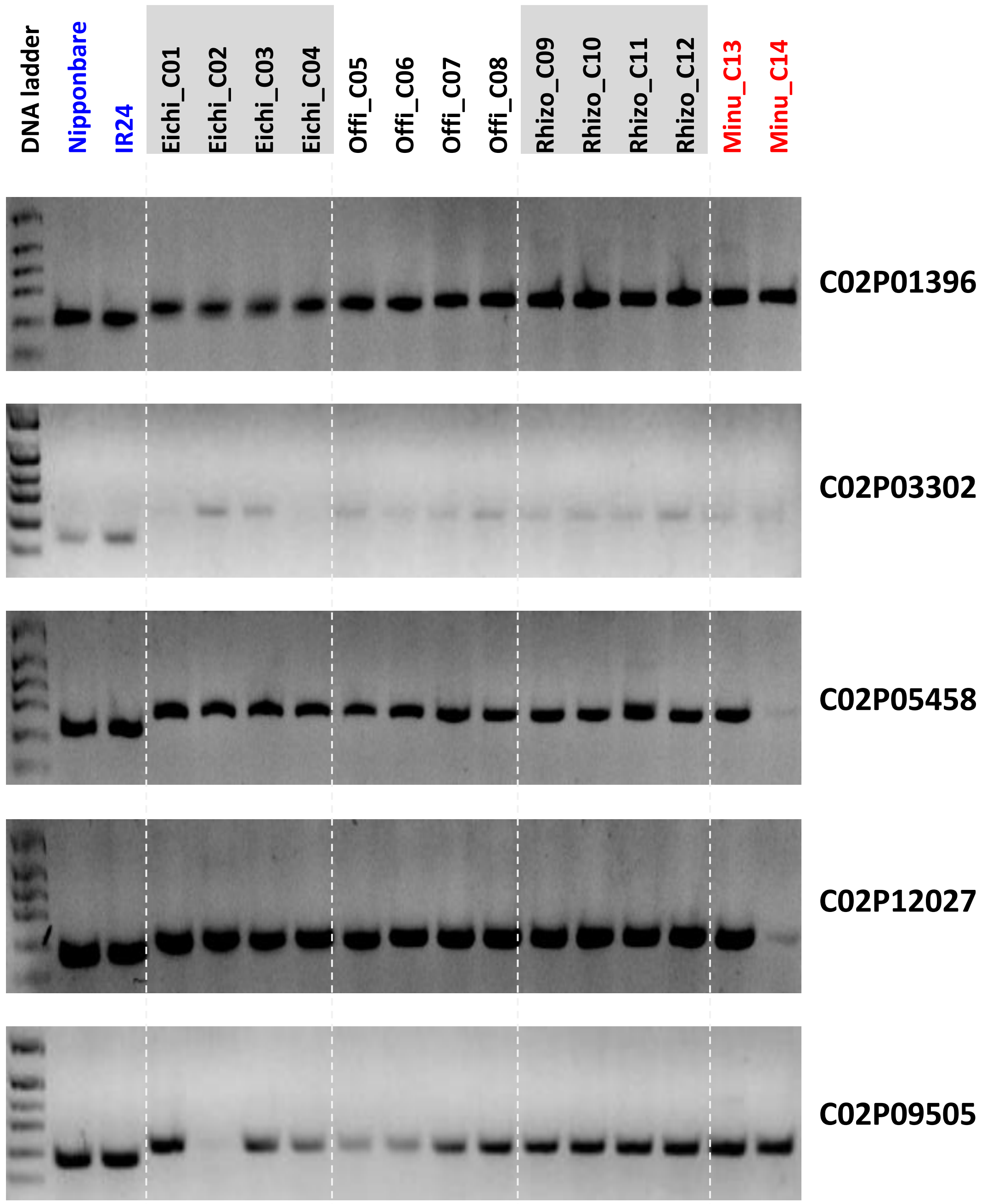

DNA ladder
Nipponbare
IR24
Eichi_C01
Eichi_C02
Eichi_C03
Eichi_C04
Offi_C05
Offi_C06
Offi_C07
Offi_C08
Rhizo_C09
Rhizo_C10
Rhizo_C11
Rhizo_C12
Minu_C13
Minu_C14
C02P01396
C02P03302
C02P05458
C02P12027
C02P09505

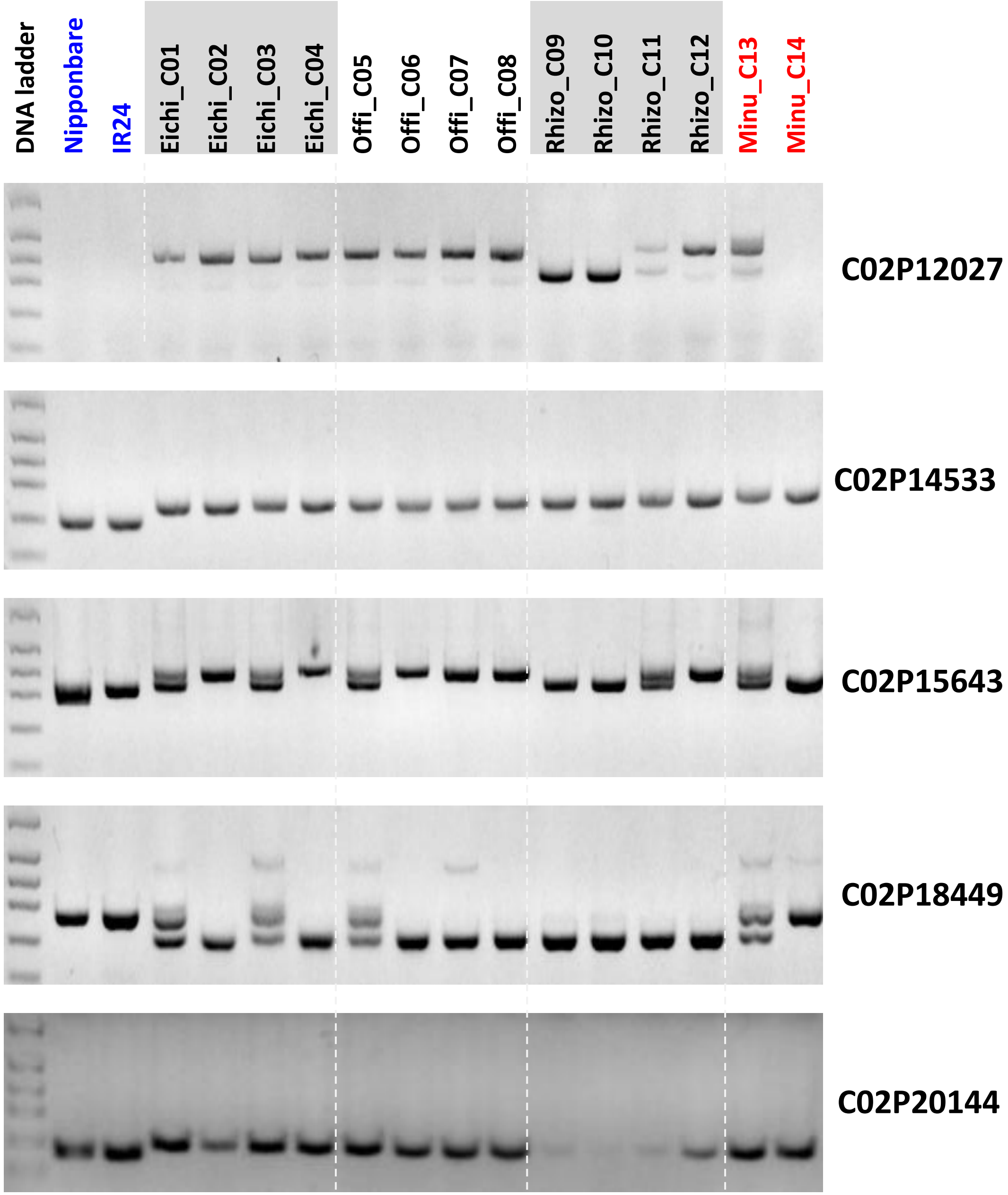
DNA ladder
Nipponbare
IR24
Eichi_C01
Eichi_C02
Eichi_C03
Eichi_C04
Offi_C05
Offi_C06
Offi_C07
Offi_C08
Rhizo_C09
Rhizo_C10
Rhizo_C11
Rhizo_C12
Minu_C13
Minu_C14
C02P12027
C02P14533
C02P15643
C02P18449
C02P20144

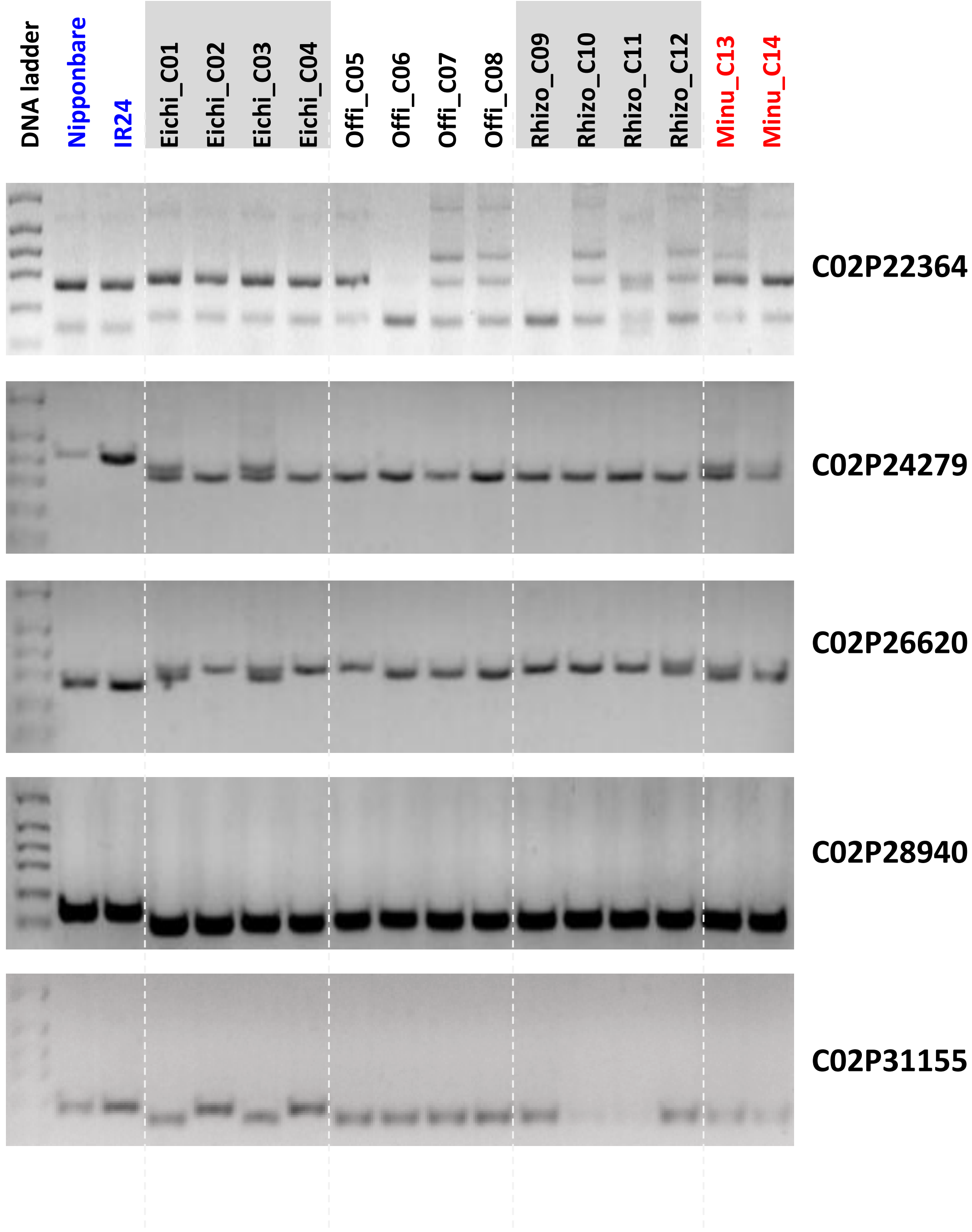

DNA ladder
Nipponbare
IR24
Eichi_C01
Eichi_C02
Eichi_C03
Eichi_C04
Offi_C05
Offi_C06
Offi_C07
Offi_C08
Rhizo_C09
Rhizo_C10
Rhizo_C11
Rhizo_C12
Minu_C13
Minu_C14
C02P22364
C02P24279
C02P26620
C02P28940
C02P31155

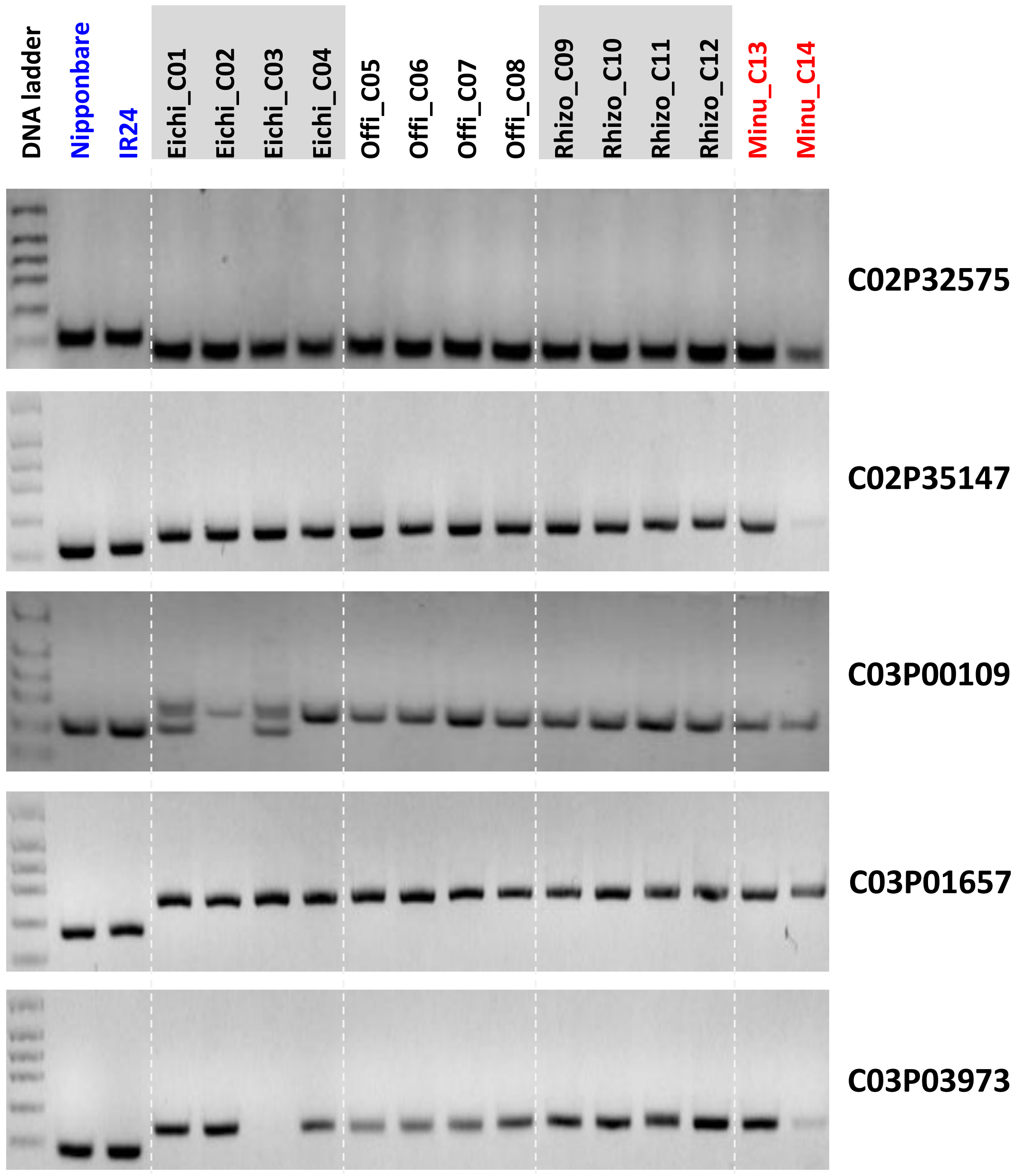
DNA ladder
Nipponbare
IR24
Eichi_C01
Eichi_C02
Eichi_C03
Eichi_C04
Offi_C05
Offi_C06
Offi_C07
Offi_C08
Rhizo_C09
Rhizo_C10
Rhizo_C11
Rhizo_C12
Minu_C13
Minu_C14
C02P32575
C02P35147
C03P00109
C03P01657
C03P03973

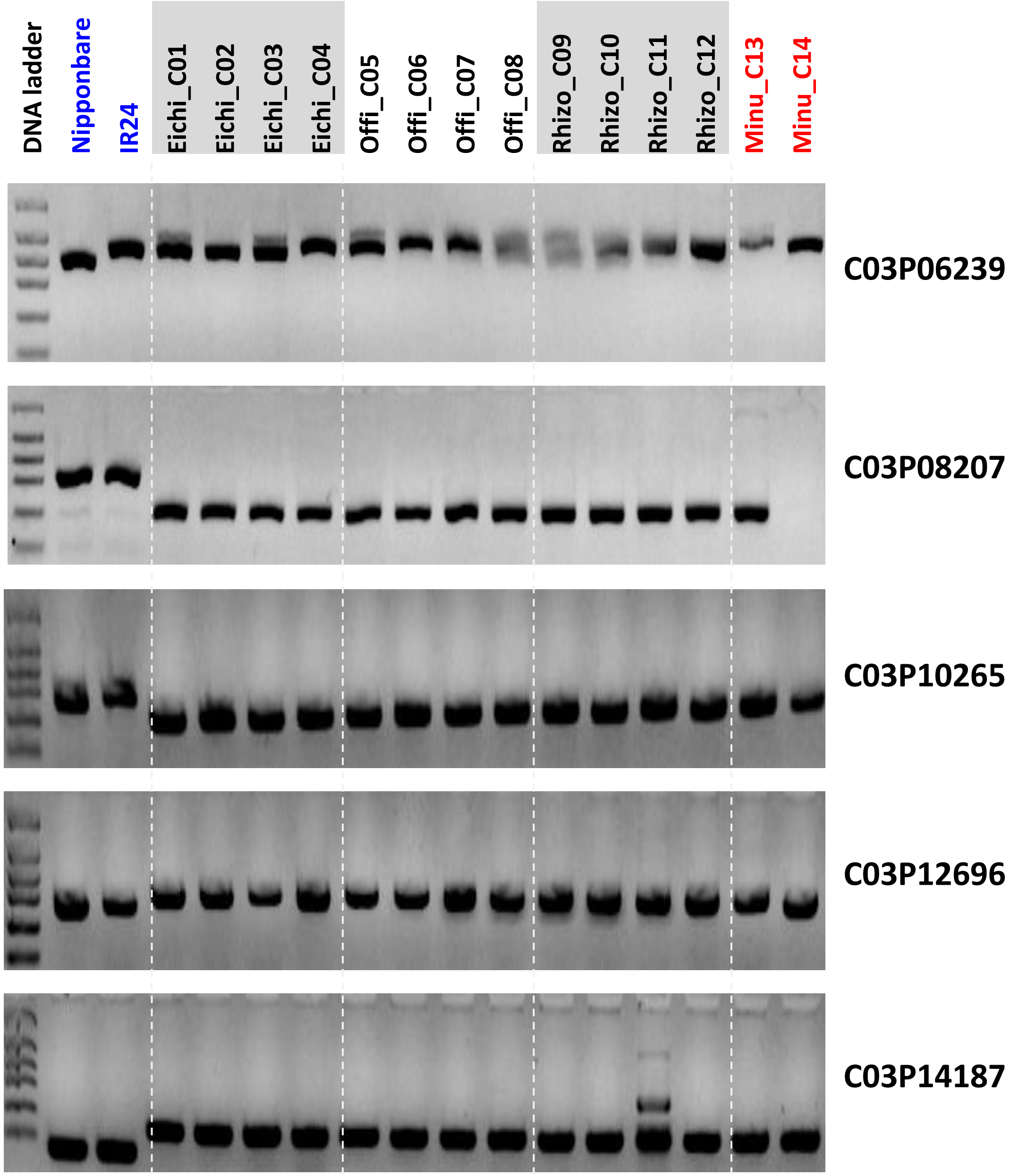
DNA ladder
Nipponbare
IR24
Eichi_C01
Eichi_C02
Eichi_C03
Eichi_C04
Offi_C05
Offi_C06
Offi_C07
Offi_C08
Rhizo_C09
Rhizo_C10
Rhizo_C11
Rhizo_C12
Minu_C13
Minu_C14
C03P06239
C03P08207
C03P10265
C03P12696
C03P14187

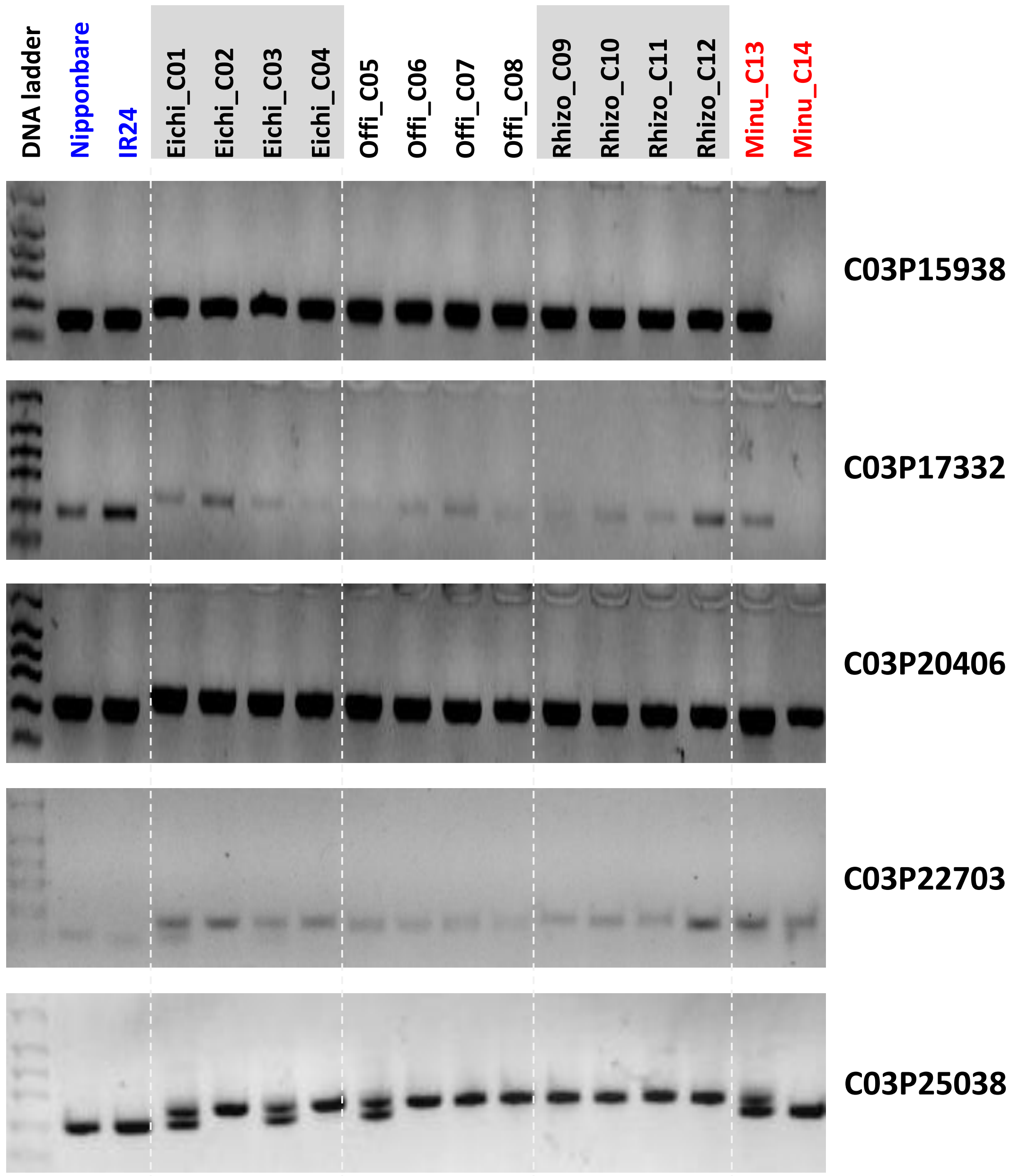

DNA ladder
Nipponbare
IR24
Eichi_C01
Eichi_C02
Eichi_C03
Eichi_C04
Offi_C05
Offi_C06
Offi_C07
Offi_C08
Rhizo_C09
Rhizo_C10
Rhizo_C11
Rhizo_C12
Minu_C13
Minu_C14
C03P15938
C03P17332
C03P20406
C03P22703
C03P25038

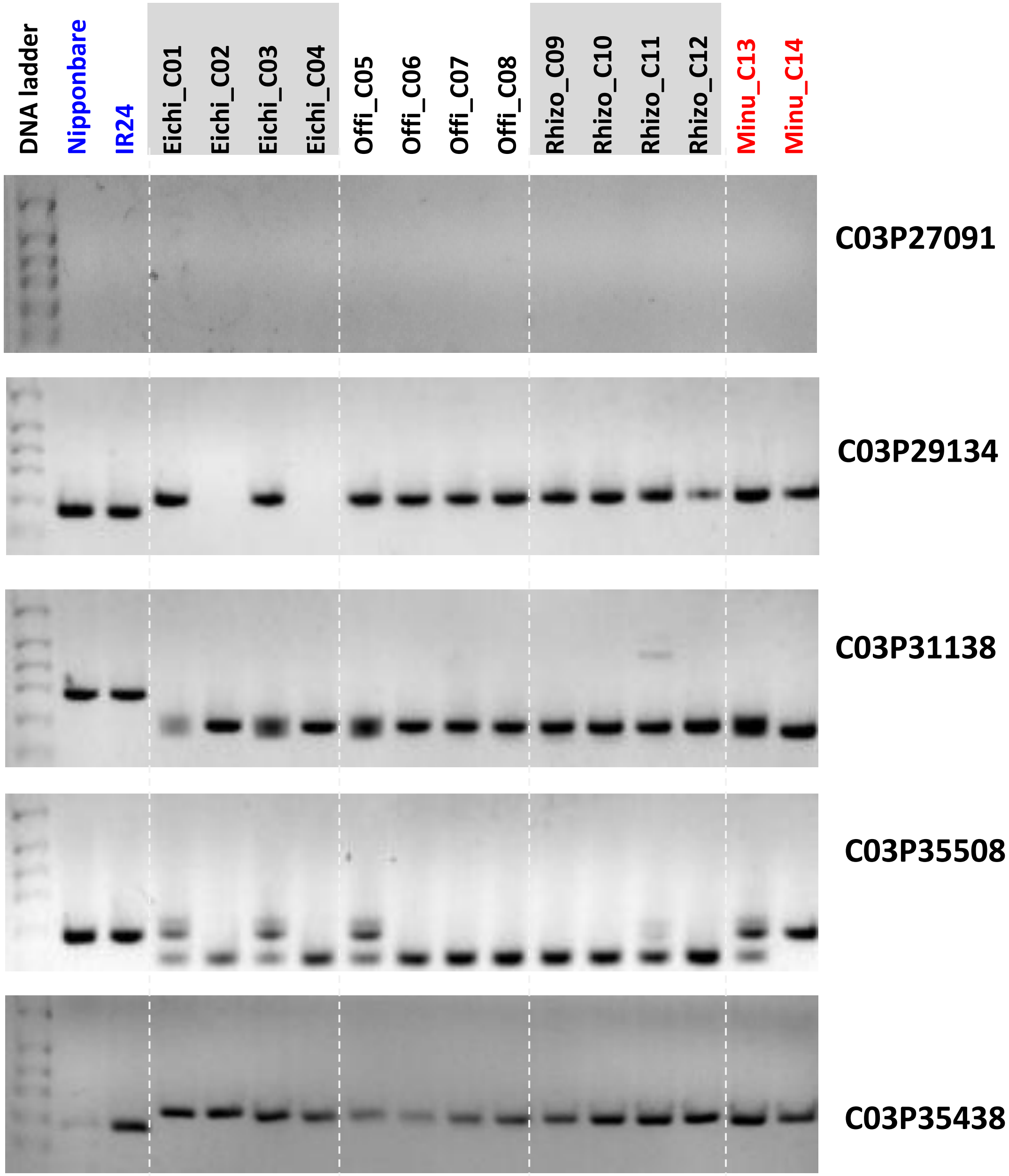

DNA ladder
Nipponbare
IR24
Eichi_C01
Eichi_C02
Eichi_C03
Eichi_C04
Offi_C05
Offi_C06
Offi_C07
Offi_C08
Rhizo_C09
Rhizo_C10
Rhizo_C11
Rhizo_C12
Minu_C13
Minu_C14
C03P27091
C03P29134
C03P31138
C03P35508
C03P35438

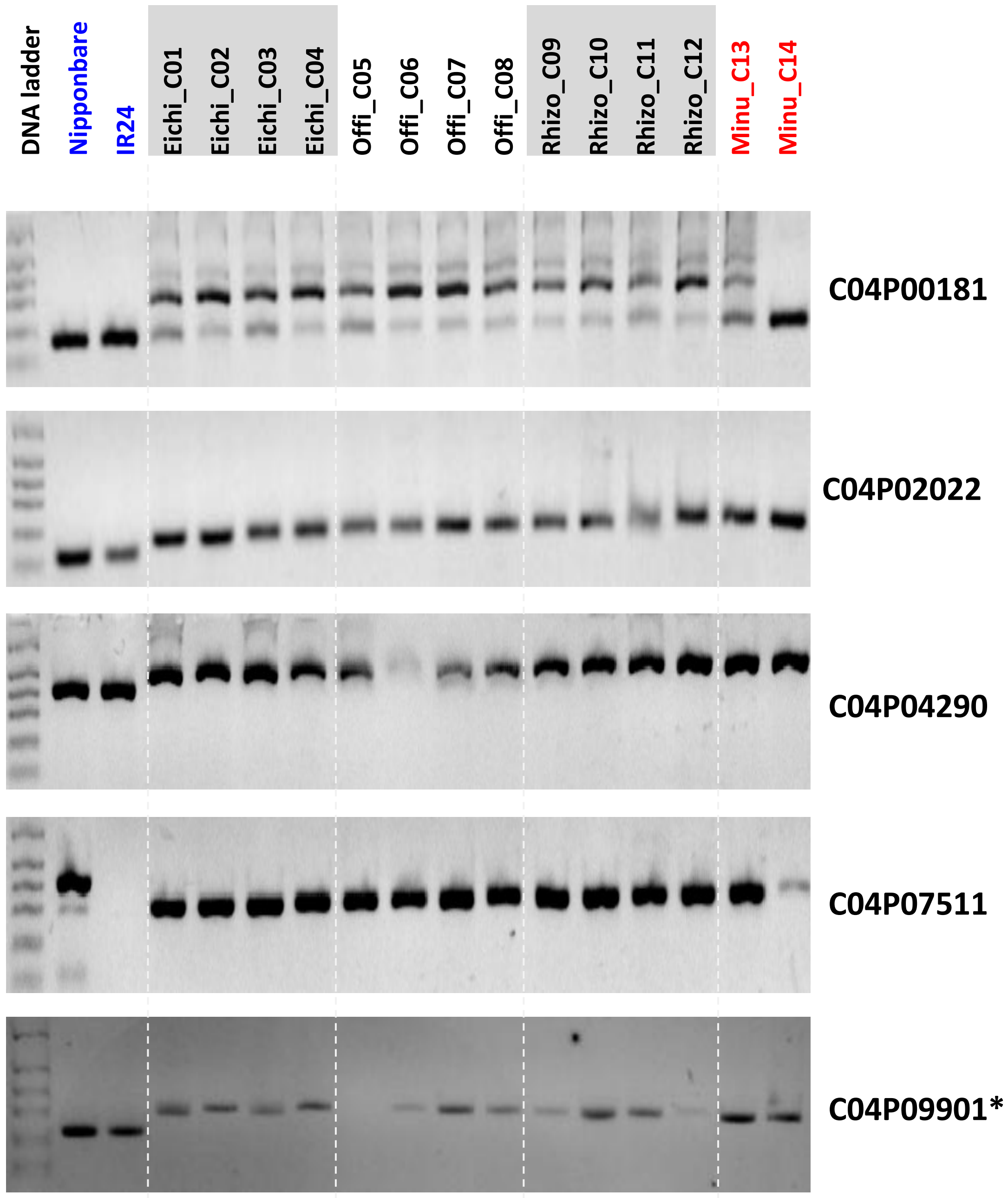
DNA ladder
Nipponbare
IR24
Eichi_C01
Eichi_C02
Eichi_C03
Eichi_C04
Offi_C05
Offi_C06
Offi_C07
Offi_C08
Rhizo_C09
Rhizo_C10
Rhizo_C11
Rhizo_C12
Minu_C13
Minu_C14
C04P00181
C04P02022
C04P04290
C04P07511
C04P09901*

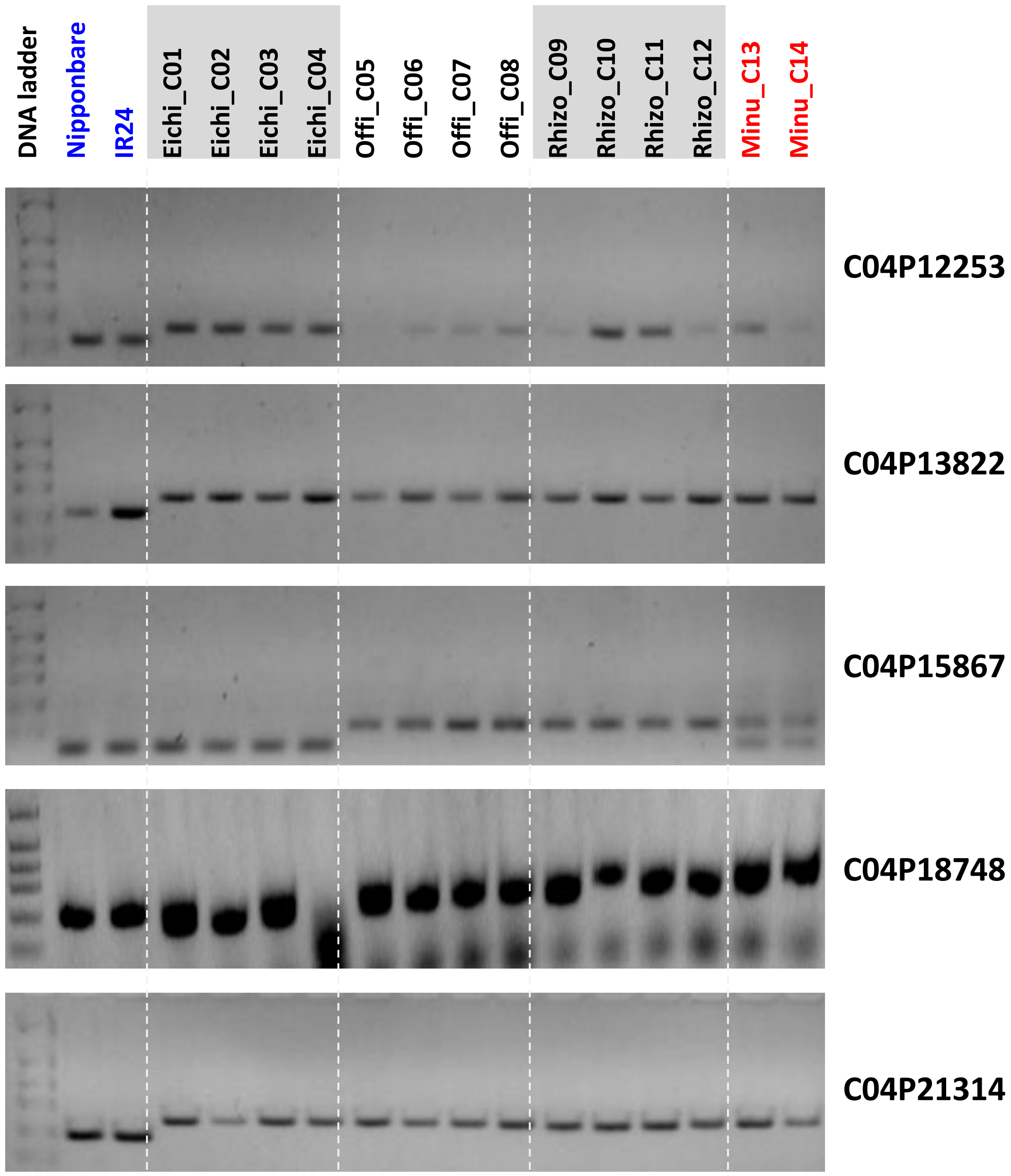
DNA ladder
Nipponbare
IR24
Eichi_C01
Eichi_C02
Eichi_C03
Eichi_C04
Offi_C05
Offi_C06
Offi_C07
Offi_C08
Rhizo_C09
Rhizo_C10
Rhizo_C11
Rhizo_C12
Minu_C13
Minu_C14
C04P12253
C04P13822
C04P15867
C04P18748
C04P21314

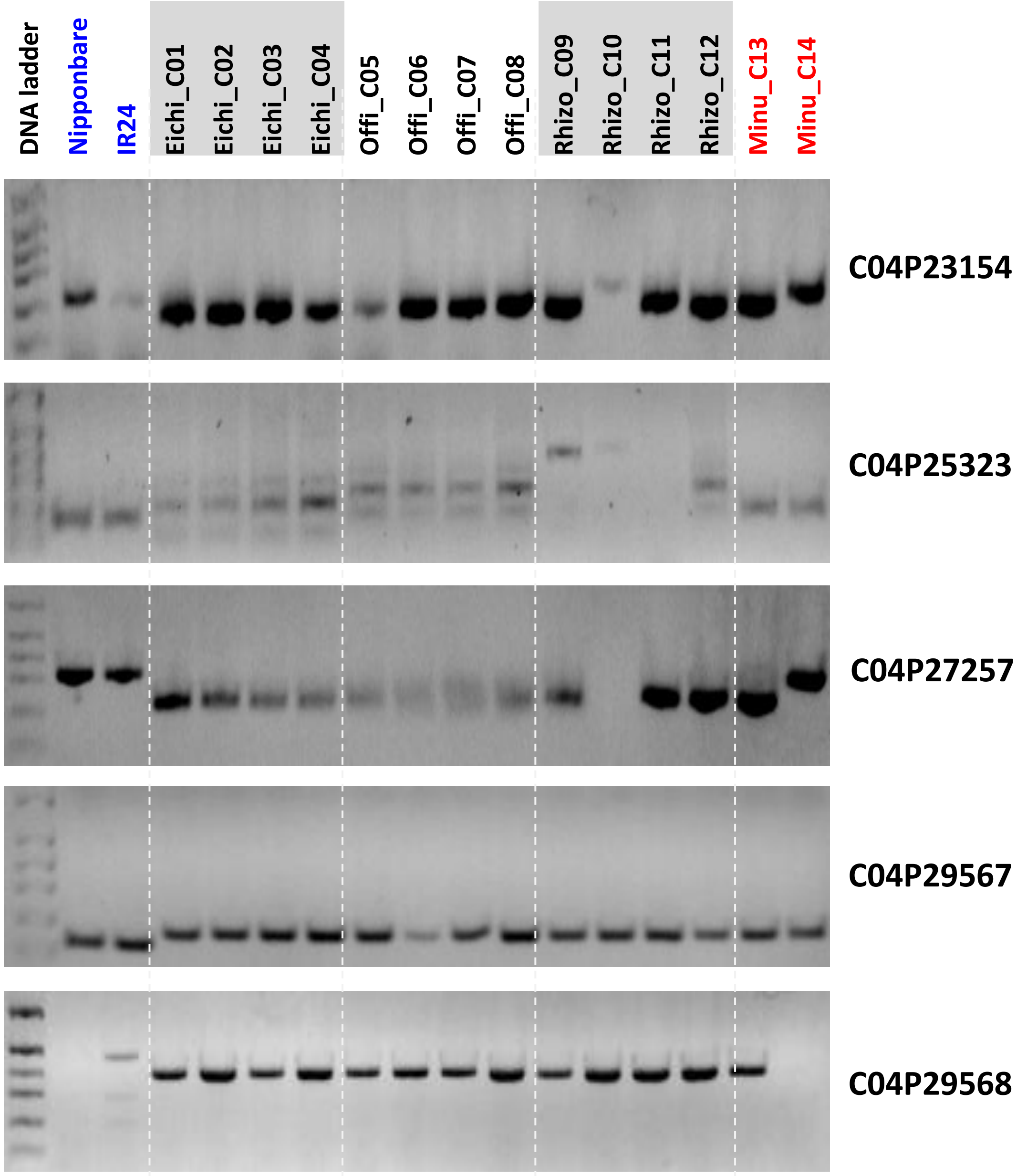
DNA ladder
Nipponbare
IR24
Eichi_C01
Eichi_C02
Eichi_C03
Eichi_C04
Offi_C05
Offi_C06
Offi_C07
Offi_C08
Rhizo_C09
Rhizo_C10
Rhizo_C11
Rhizo_C12
Minu_C13
Minu_C14
C04P23154
C04P25323
C04P27257
C04P29567
C04P29568

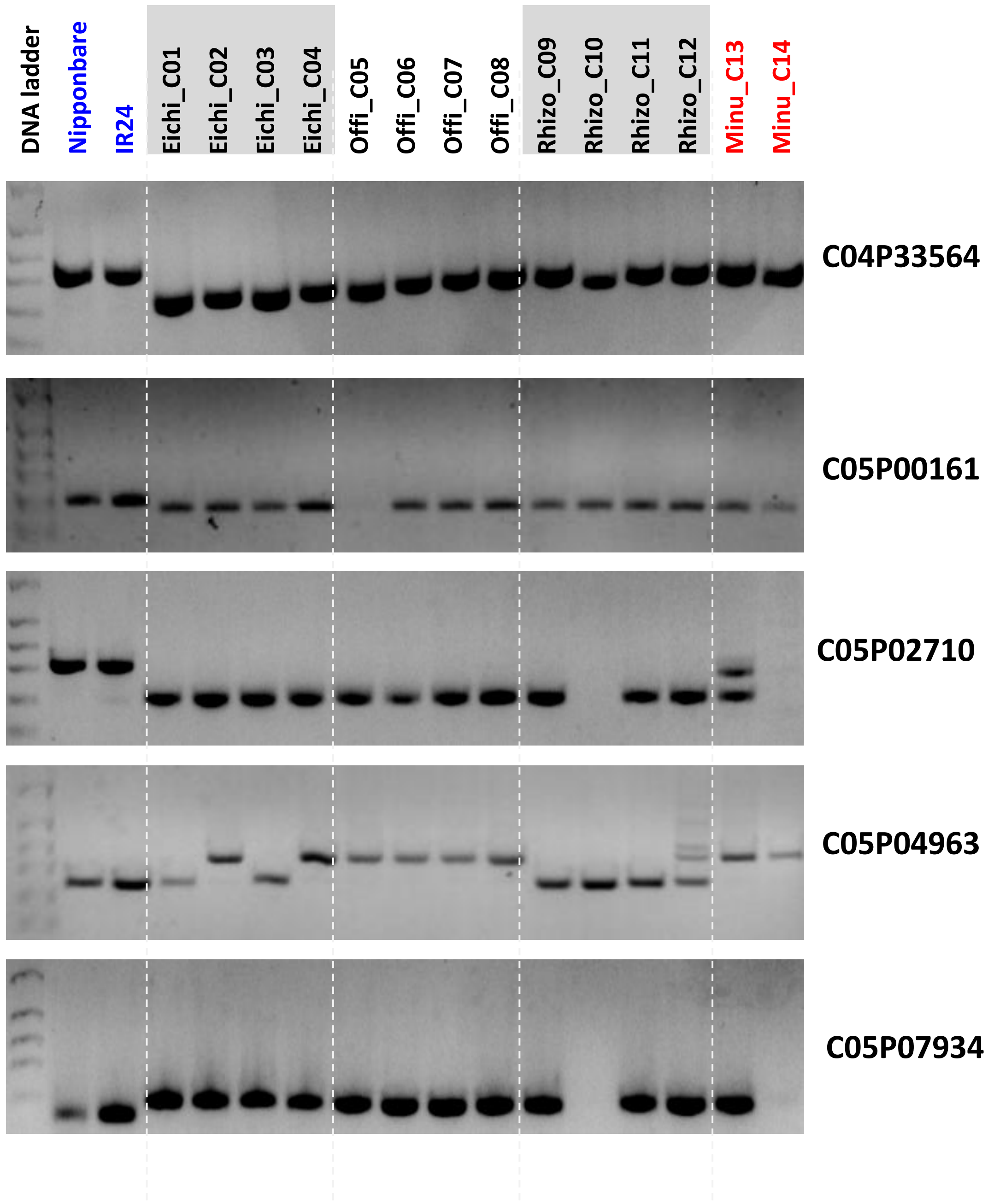

DNA ladder
Nipponbare
IR24
Eichi_C01
Eichi_C02
Eichi_C03
Eichi_C04
Offi_C05
Offi_C06
Offi_C07
Offi_C08
Rhizo_C09
Rhizo_C10
Rhizo_C11
Rhizo_C12
Minu_C13
Minu_C14
C04P33564
C05P00161
C05P02710
C05P04963
C05P07934

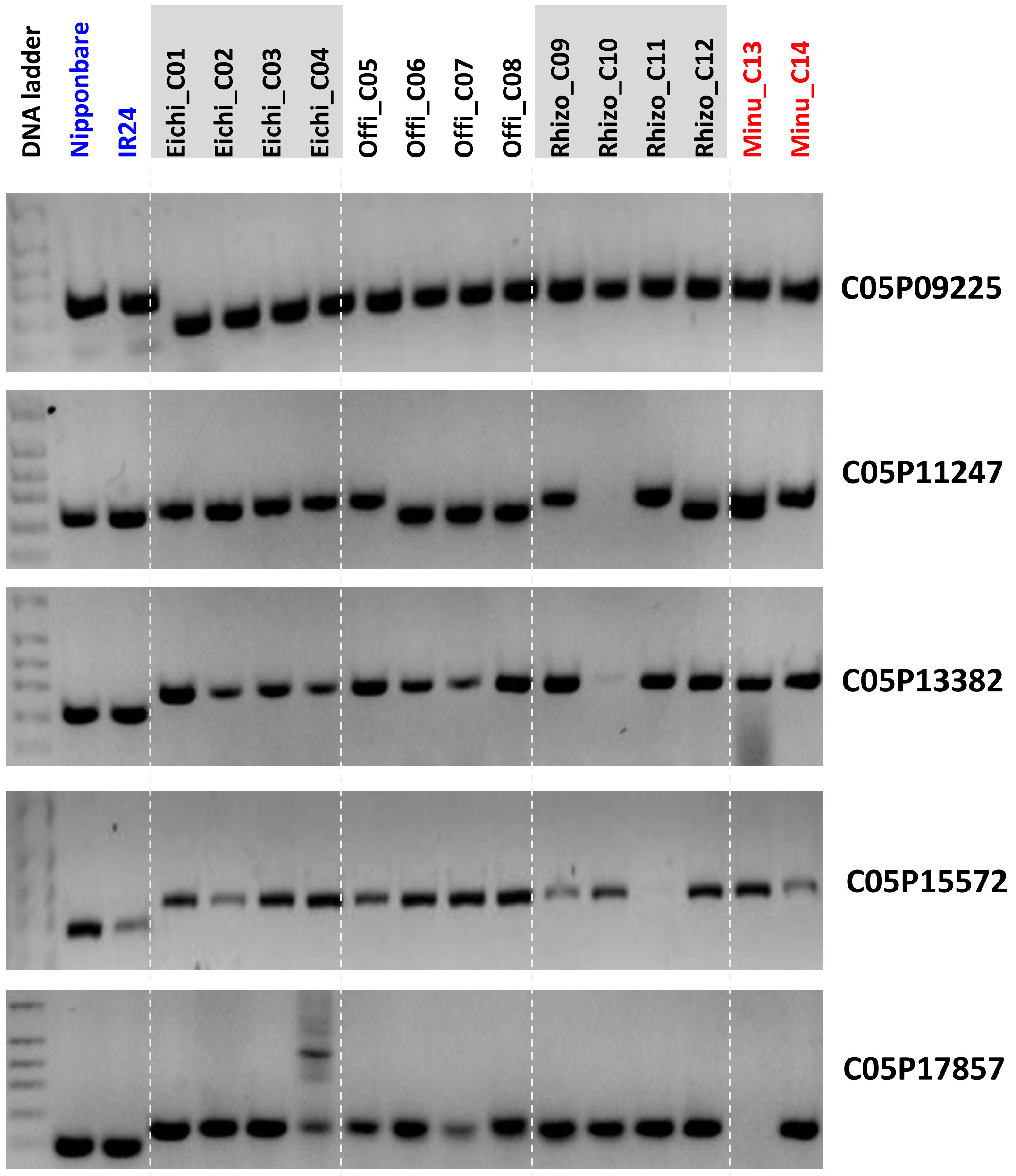

DNA ladder
Nipponbare
IR24
Eichi_C01
Eichi_C02
Eichi_C03
Eichi_C04
Offi_C05
Offi_C06
Offi_C07
Offi_C08
Rhizo_C09
Rhizo_C10
Rhizo_C11
Rhizo_C12
Minu_C13
Minu_C14
C05P09225
C05P11247
C05P13382
C05P15572
C05P17857

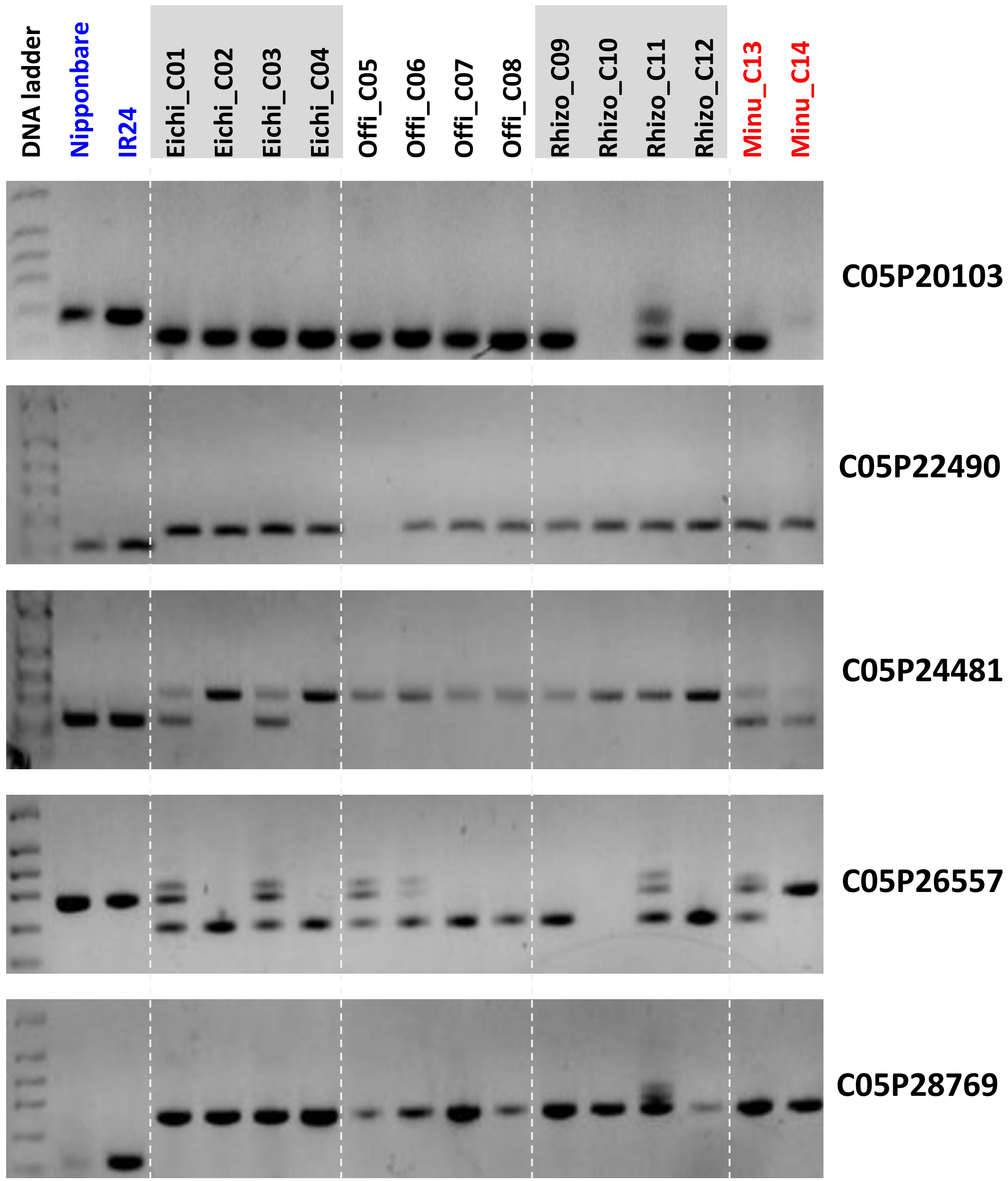
DNA ladder
Nipponbare
IR24
Eichi_C01
Eichi_C02
Eichi_C03
Eichi_C04
Offi_C05
Offi_C06
Offi_C07
Offi_C08
Rhizo_C09
Rhizo_C10
Rhizo_C11
Rhizo_C12
Minu_C13
Minu_C14
C05P20103
C05P22490
C05P24481
C05P26557
C05P28769

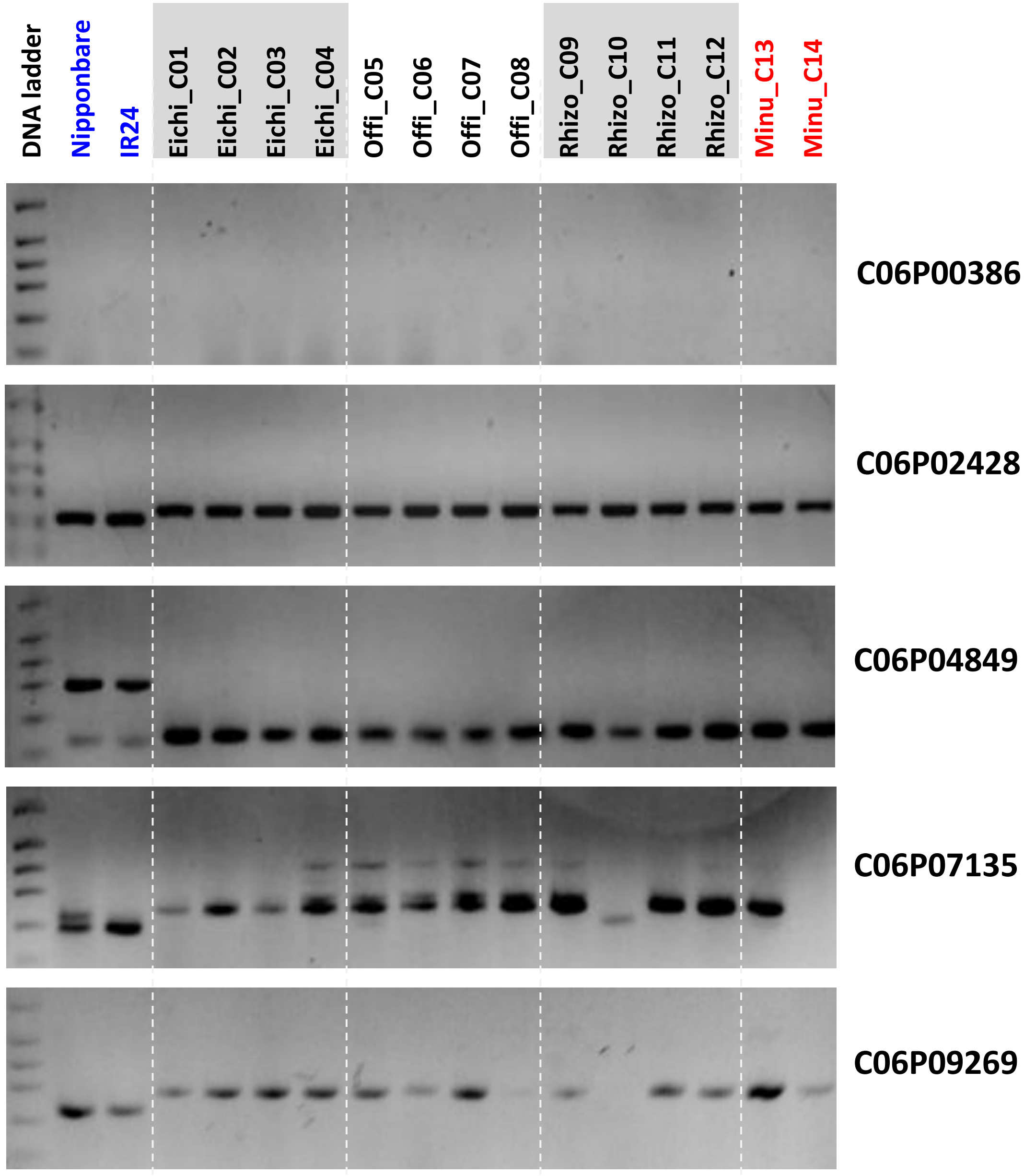
DNA ladder
Nipponbare
IR24
Eichi_C01
Eichi_C02
Eichi_C03
Eichi_C04
Offi_C05
Offi_C06
Offi_C07
Offi_C08
Rhizo_C09
Rhizo_C10
Rhizo_C11
Rhizo_C12
Minu_C13
Minu_C14
C06P00386
C06P02428
C06P04849
C06P07135
C06P09269

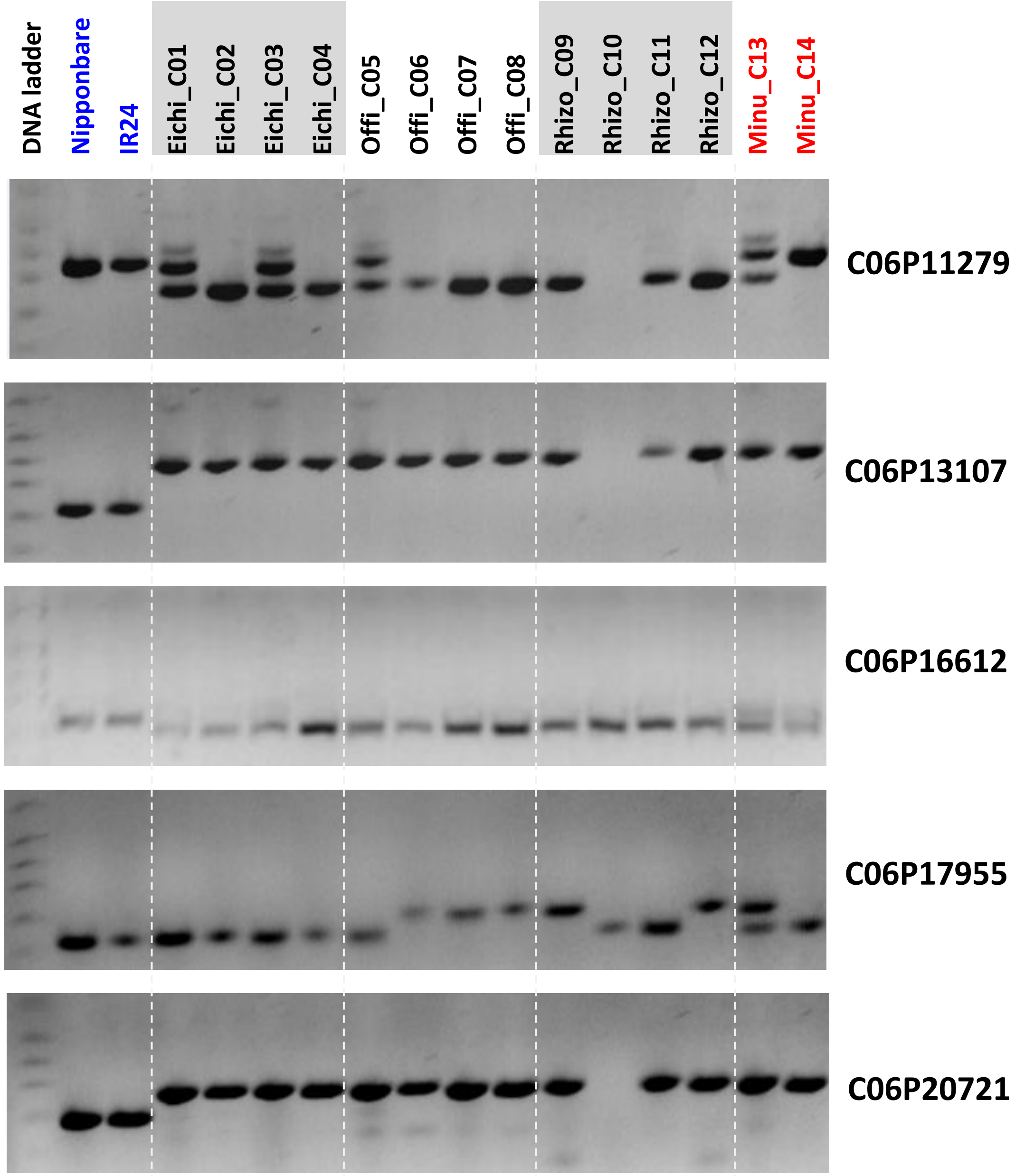
DNA ladder
Nipponbare
IR24
Eichi_C01
Eichi_C02
Eichi_C03
Eichi_C04
Offi_C05
Offi_C06
Offi_C07
Offi_C08
Rhizo_C09
Rhizo_C10
Rhizo_C11
Rhizo_C12
Minu_C13
Minu_C14
C06P11279
C06P13107
C06P16612
C06P17955
C06P20721

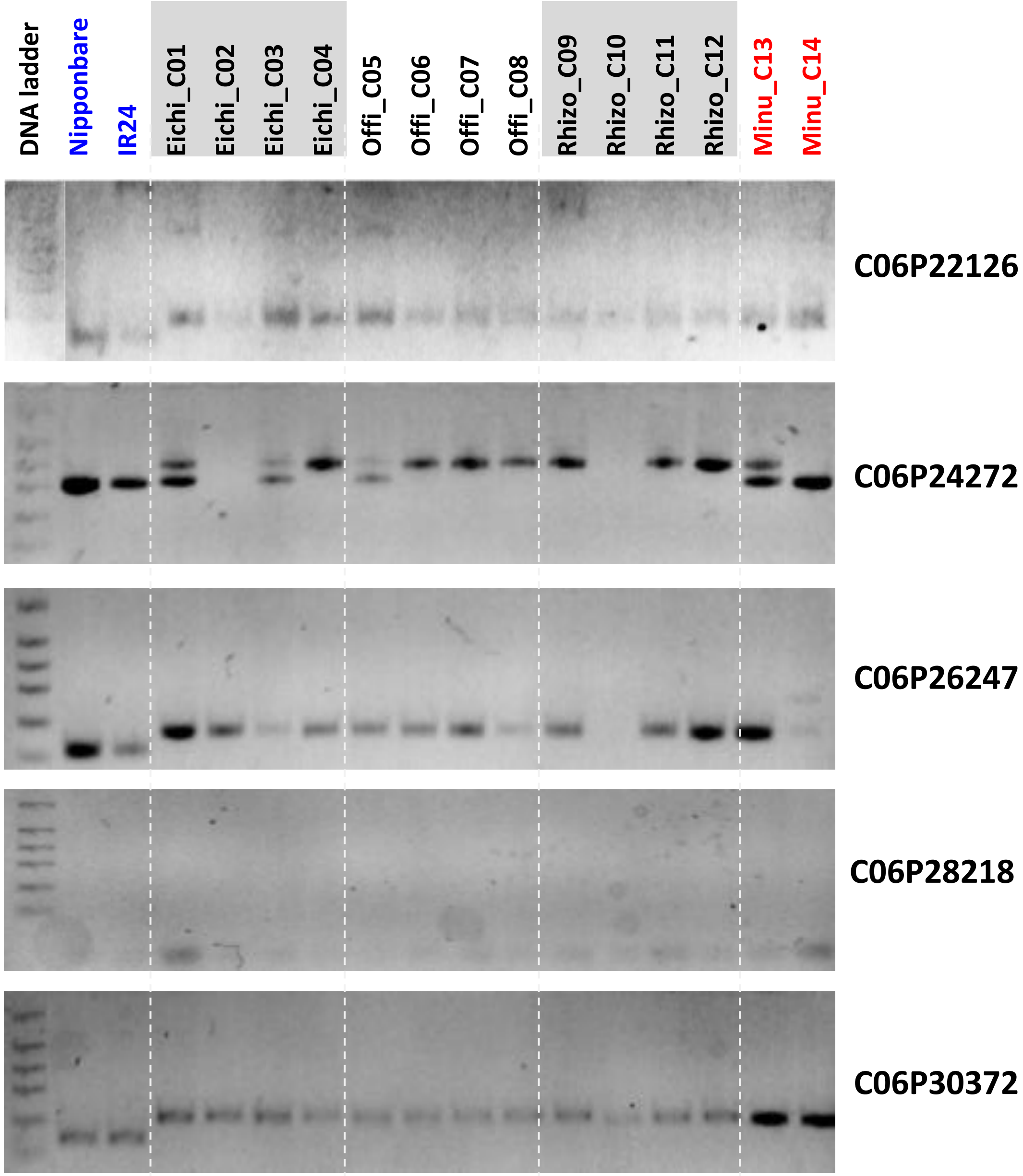
DNA ladder
Nipponbare
IR24
Eichi_C01
Eichi_C02
Eichi_C03
Eichi_C04
Offi_C05
Offi_C06
Offi_C07
Offi_C08
Rhizo_C09
Rhizo_C10
Rhizo_C11
Rhizo_C12
Minu_C13
Minu_C14
C06P22126
C06P24272
C06P26247
C06P28218
C06P30372

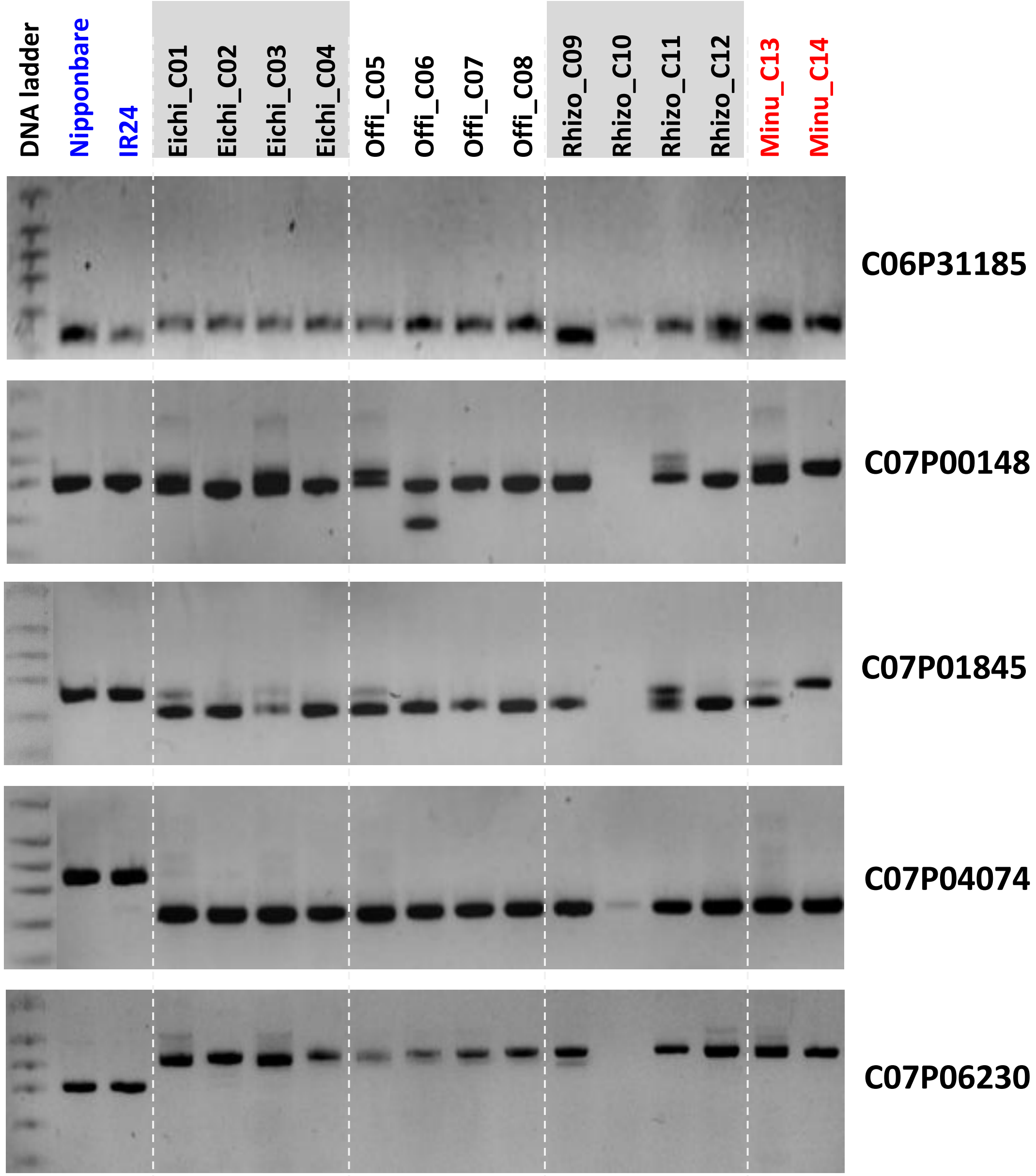

DNA ladder
Nipponbare
IR24
Eichi_C01
Eichi_C02
Eichi_C03
Eichi_C04
Offi_C05
Offi_C06
Offi_C07
Offi_C08
Rhizo_C09
Rhizo_C10
Rhizo_C11
Rhizo_C12
Minu_C13
Minu_C14
C06P31185
C07P00148
C07P01845
C07P04074
C07P06230

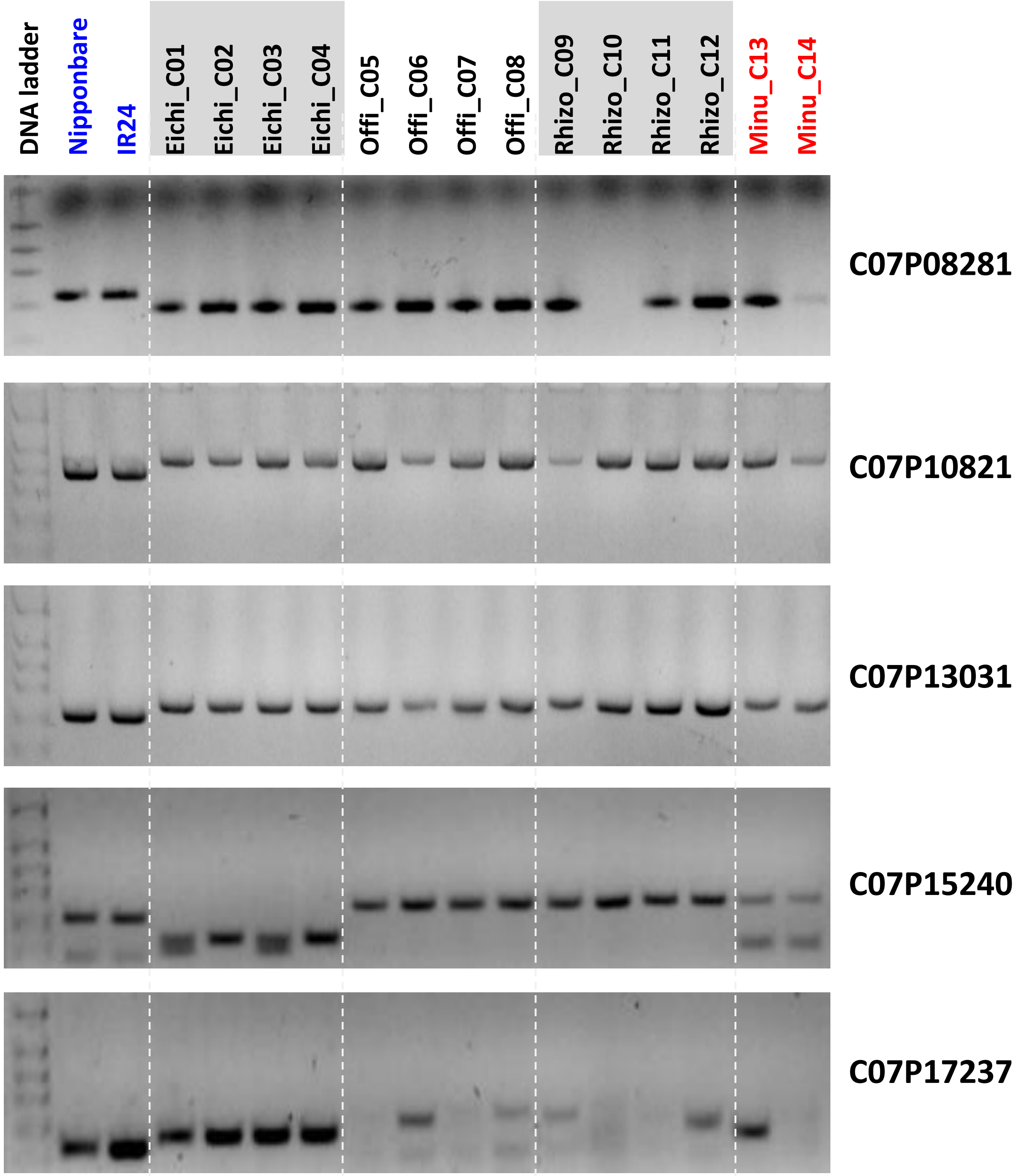
DNA ladder
Nipponbare
IR24
Eichi_C01
Eichi_C02
Eichi_C03
Eichi_C04
Offi_C05
Offi_C06
Offi_C07
Offi_C08
Rhizo_C09
Rhizo_C10
Rhizo_C11
Rhizo_C12
Minu_C13
Minu_C14
C07P08281
C07P10821
C07P13031
C07P15240
C07P17237

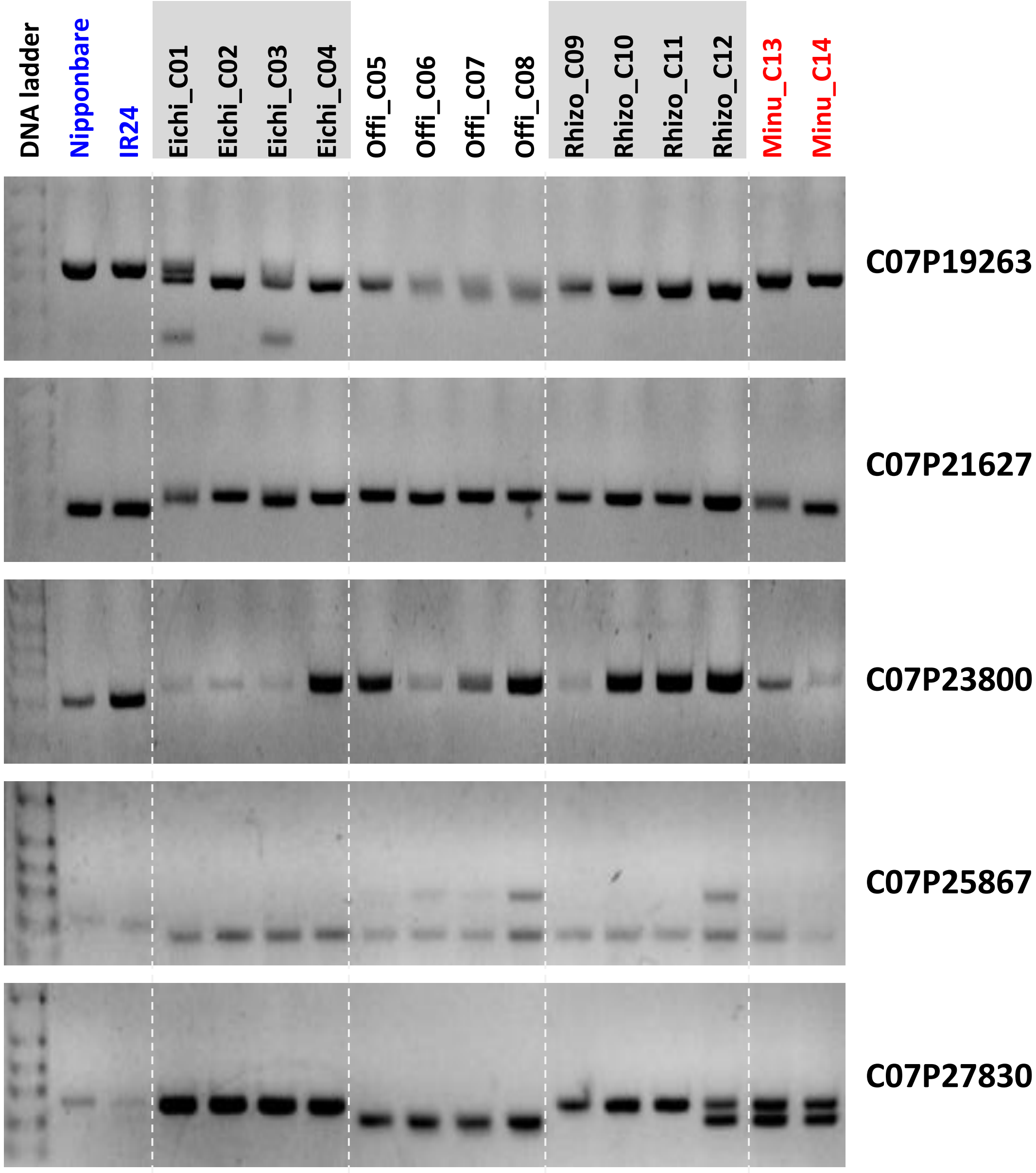
DNA ladder
Nipponbare
IR24
Eichi_C01
Eichi_C02
Eichi_C03
Eichi_C04
Offi_C05
Offi_C06
Offi_C07
Offi_C08
Rhizo_C09
Rhizo_C10
Rhizo_C11
Rhizo_C12
Minu_C13
Minu_C14
C07P19263
C07P21627
C07P23800
C07P25867
C07P27830

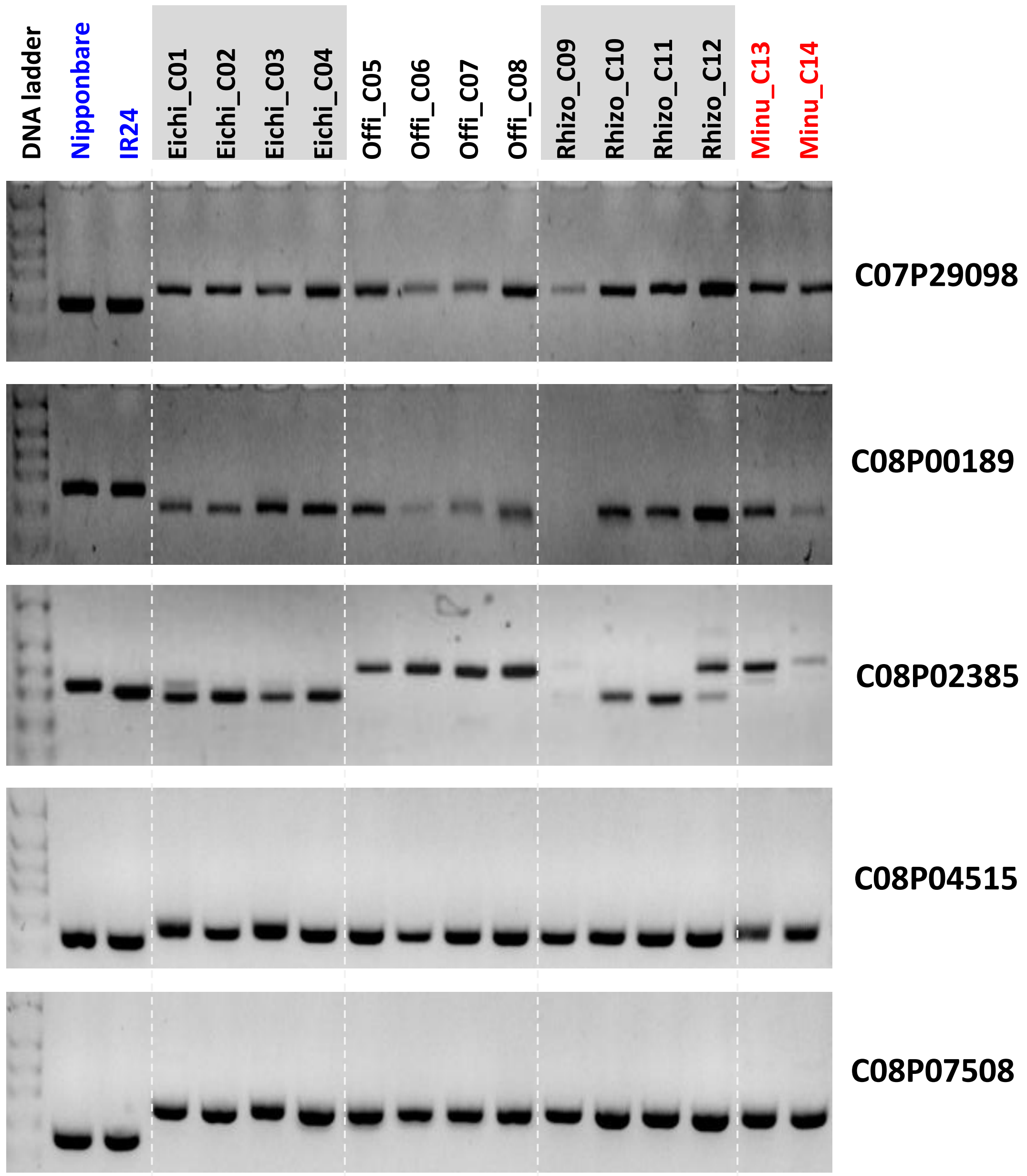
DNA ladder
Nipponbare
IR24
Eichi_C01
Eichi_C02
Eichi_C03
Eichi_C04
Offi_C05
Offi_C06
Offi_C07
Offi_C08
Rhizo_C09
Rhizo_C10
Rhizo_C11
Rhizo_C12
Minu_C13
Minu_C14
C07P29098
C08P00189
C08P02385
C08P04515
C08P07508

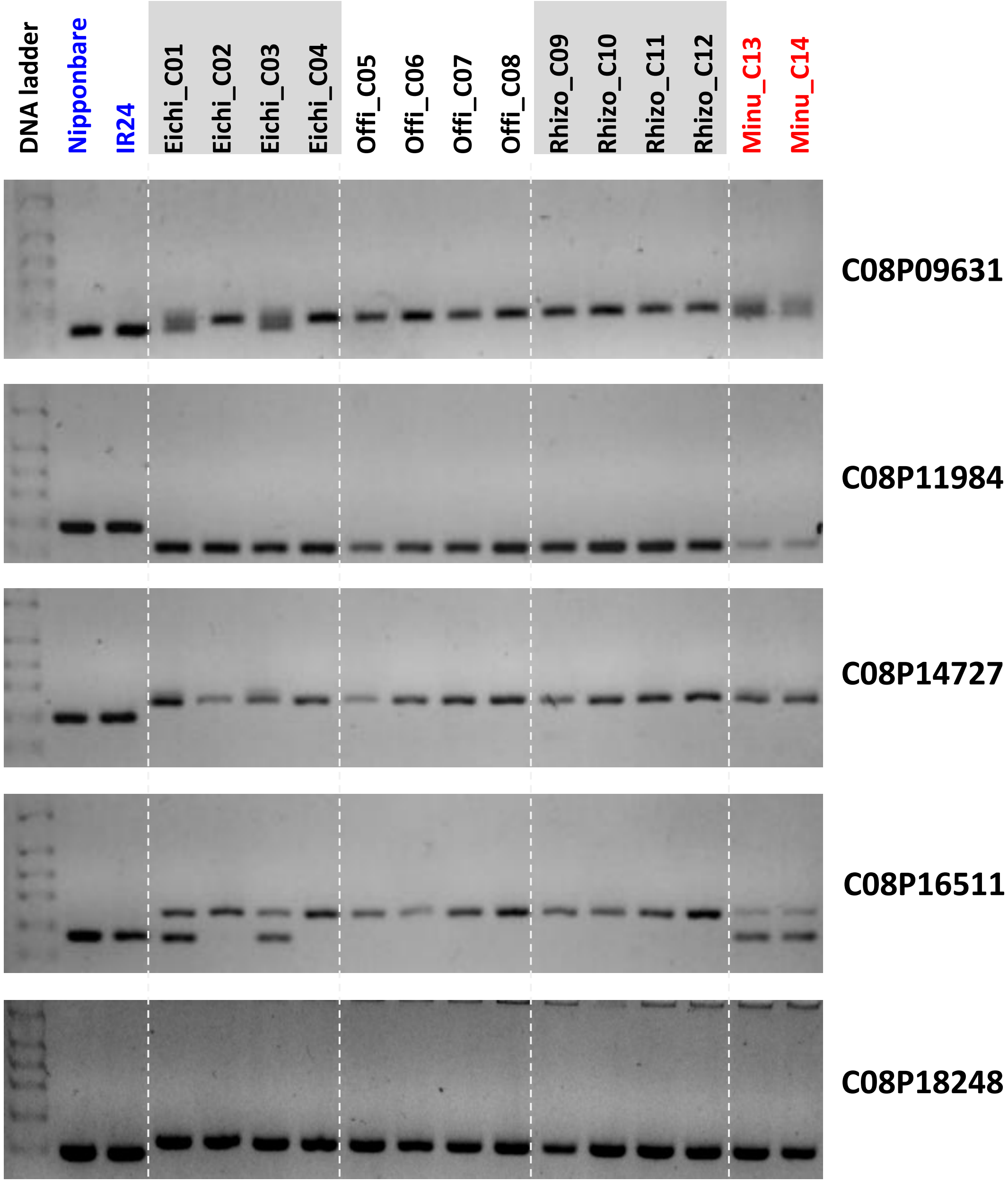
DNA ladder
Nipponbare
IR24
Eichi_C01
Eichi_C02
Eichi_C03
Eichi_C04
Offi_C05
Offi_C06
Offi_C07
Offi_C08
Rhizo_C09
Rhizo_C10
Rhizo_C11
Rhizo_C12
Minu_C13
Minu_C14
C08P09631
C08P11984
C08P14727
C08P16511
C08P18248

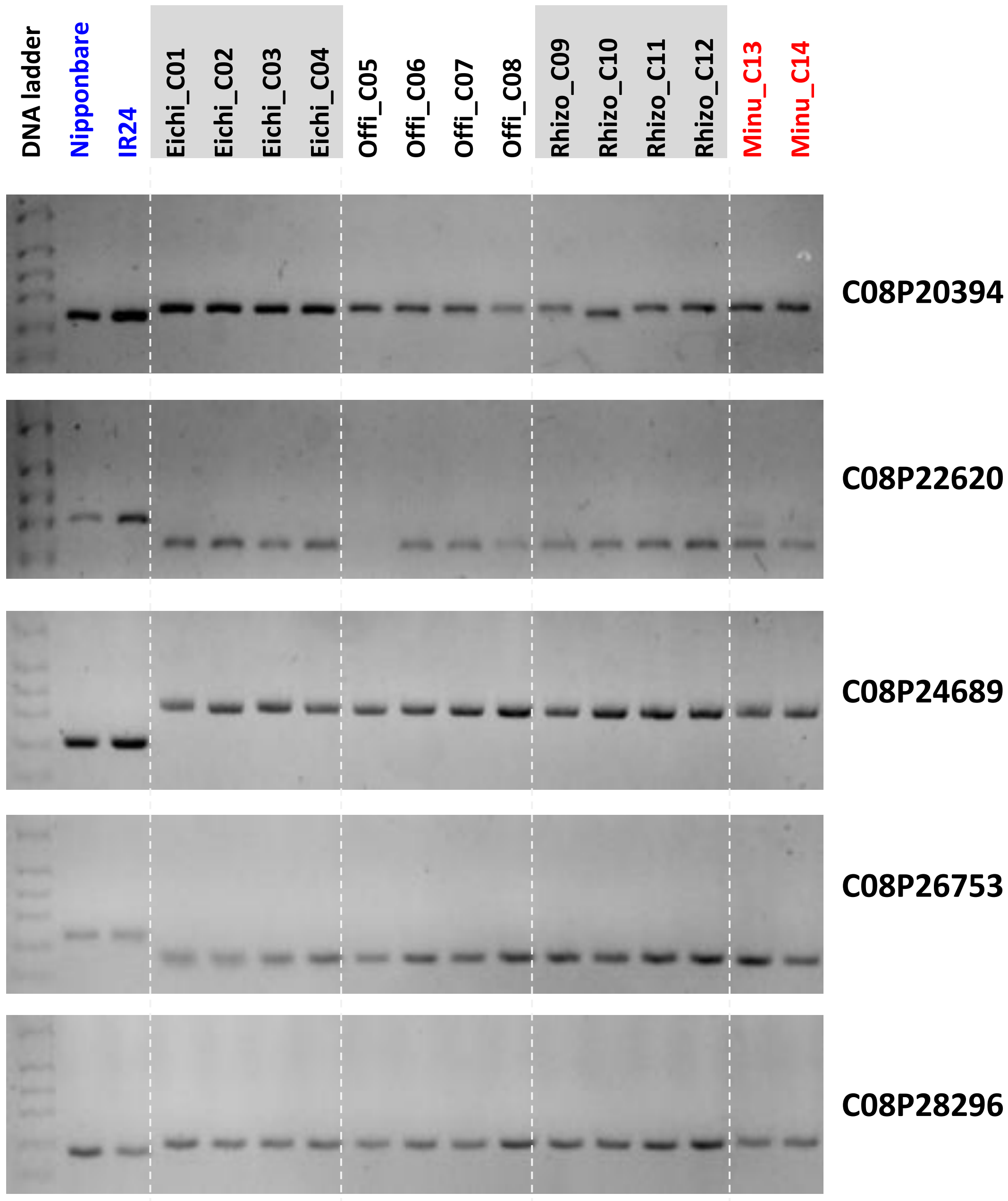
DNA ladder
Nipponbare
IR24
Eichi_C01
Eichi_C02
Eichi_C03
Eichi_C04
Offi_C05
Offi_C06
Offi_C07
Offi_C08
Rhizo_C09
Rhizo_C10
Rhizo_C11
Rhizo_C12
Minu_C13
Minu_C14
C08P20394
C08P22620
C08P24689
C08P26753
C08P28296

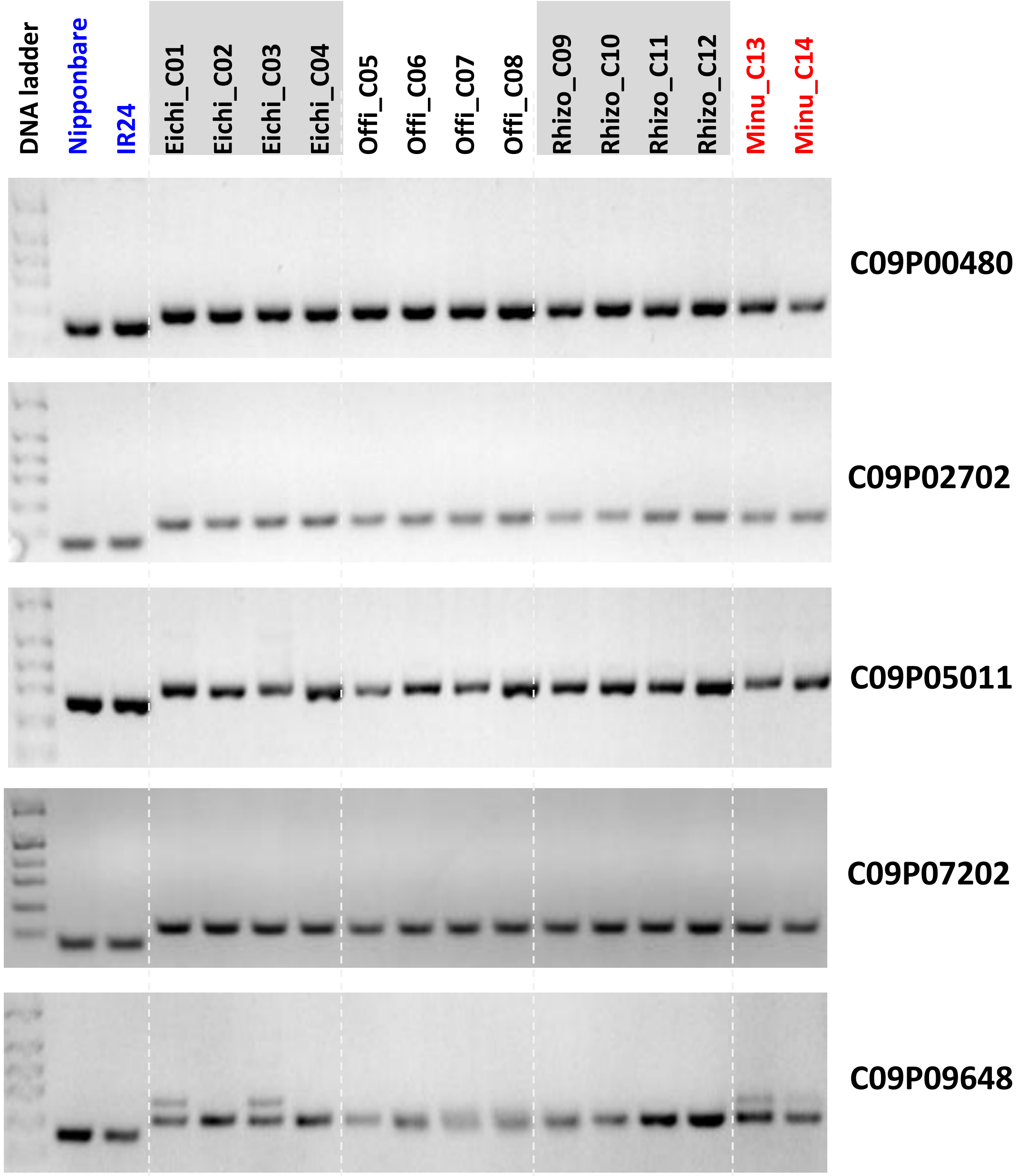
DNA ladder
Nipponbare
IR24
Eichi_C01
Eichi_C02
Eichi_C03
Eichi_C04
Offi_C05
Offi_C06
Offi_C07
Offi_C08
Rhizo_C09
Rhizo_C10
Rhizo_C11
Rhizo_C12
Minu_C13
Minu_C14
C09P00480
C09P02702
C09P05011
C09P07202
C09P09648

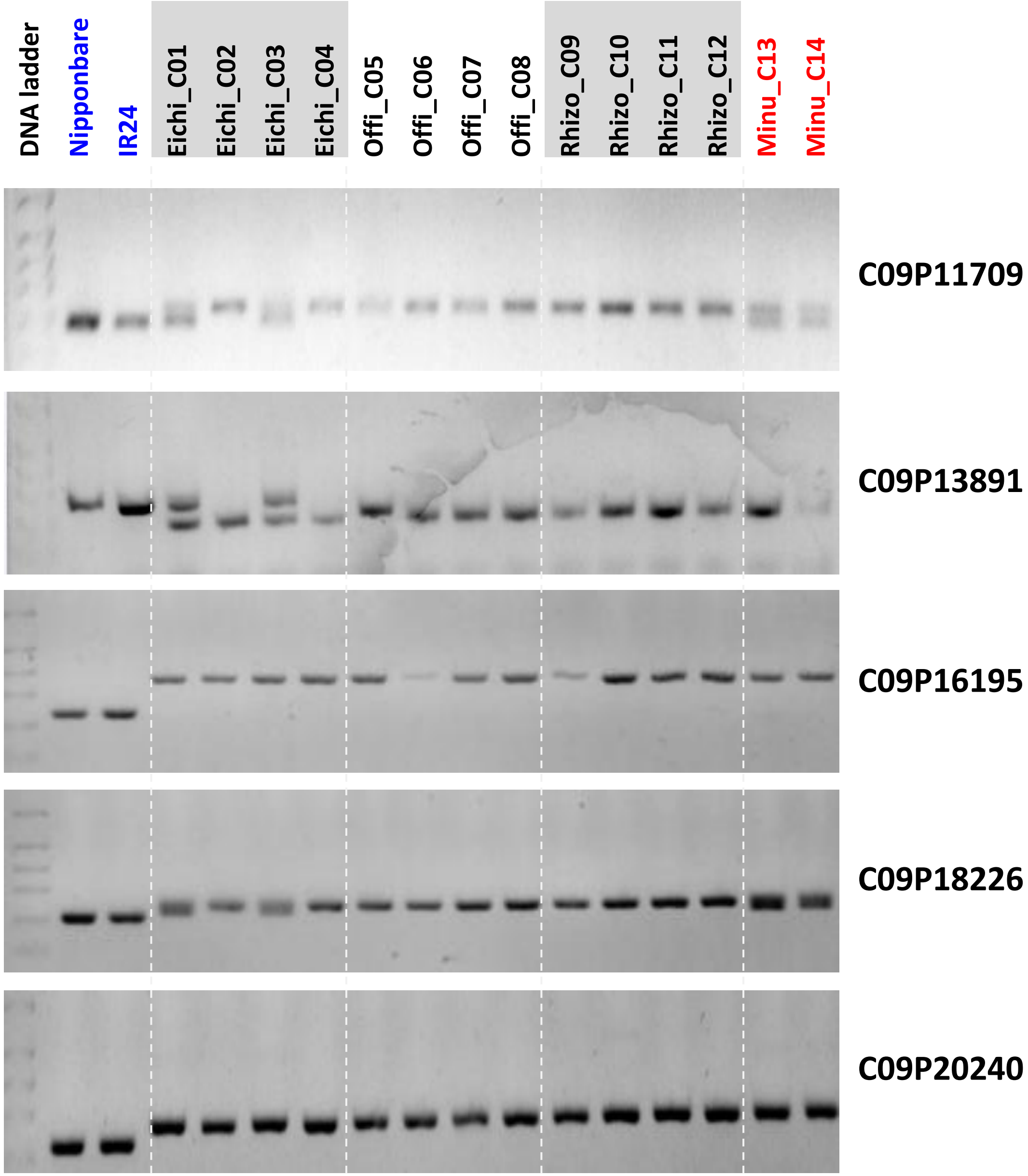

DNA ladder
Nipponbare
IR24
Eichi_C01
Eichi_C02
Eichi_C03
Eichi_C04
Offi_C05
Offi_C06
Offi_C07
Offi_C08
Rhizo_C09
Rhizo_C10
Rhizo_C11
Rhizo_C12
Minu_C13
Minu_C14
C09P11709
C09P13891
C09P16195
C09P18226
C09P20240

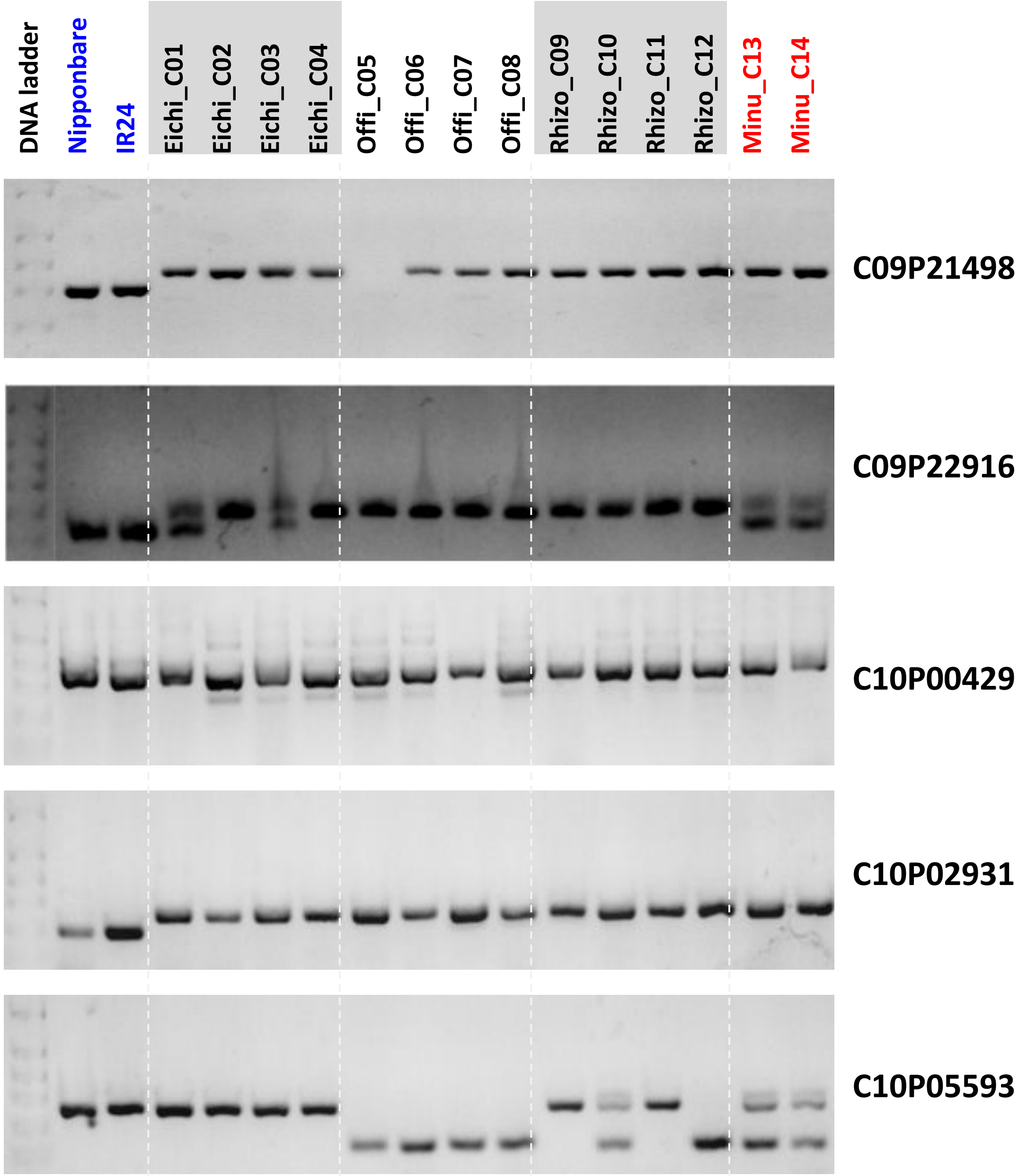
DNA ladder
Nipponbare
IR24
Eichi_C01
Eichi_C02
Eichi_C03
Eichi_C04
Offi_C05
Offi_C06
Offi_C07
Offi_C08
Rhizo_C09
Rhizo_C10
Rhizo_C11
Rhizo_C12
Minu_C13
Minu_C14
C09P21498
C09P22916
C10P00429
C10P02931
C10P05593

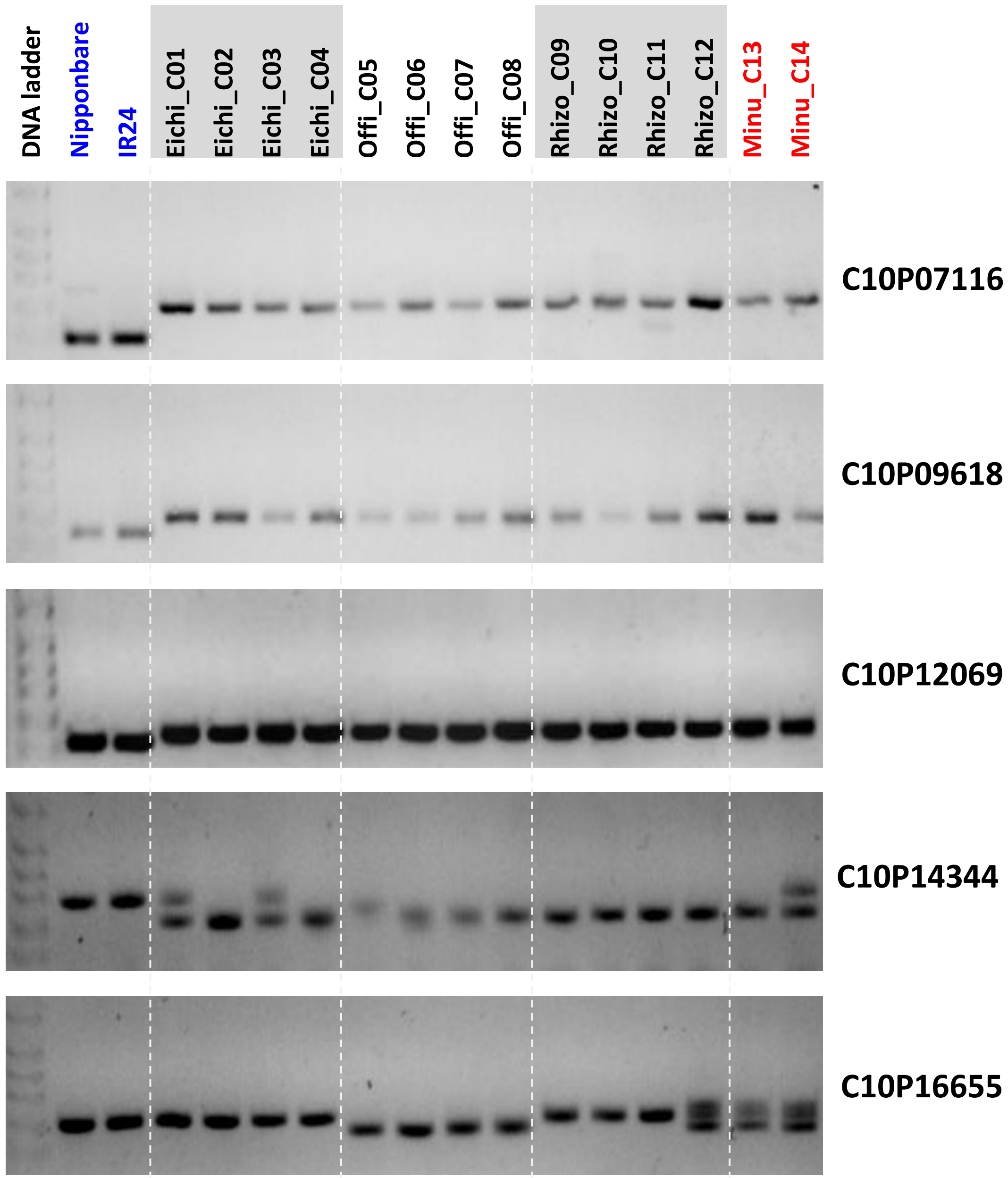
DNA ladder
Nipponbare
IR24
Eichi_C01
Eichi_C02
Eichi_C03
Eichi_C04
Offi_C05
Offi_C06
Offi_C07
Offi_C08
Rhizo_C09
Rhizo_C10
Rhizo_C11
Rhizo_C12
Minu_C13
Minu_C14
C10P07116
C10P09618
C10P12069
C10P14344
C10P16655

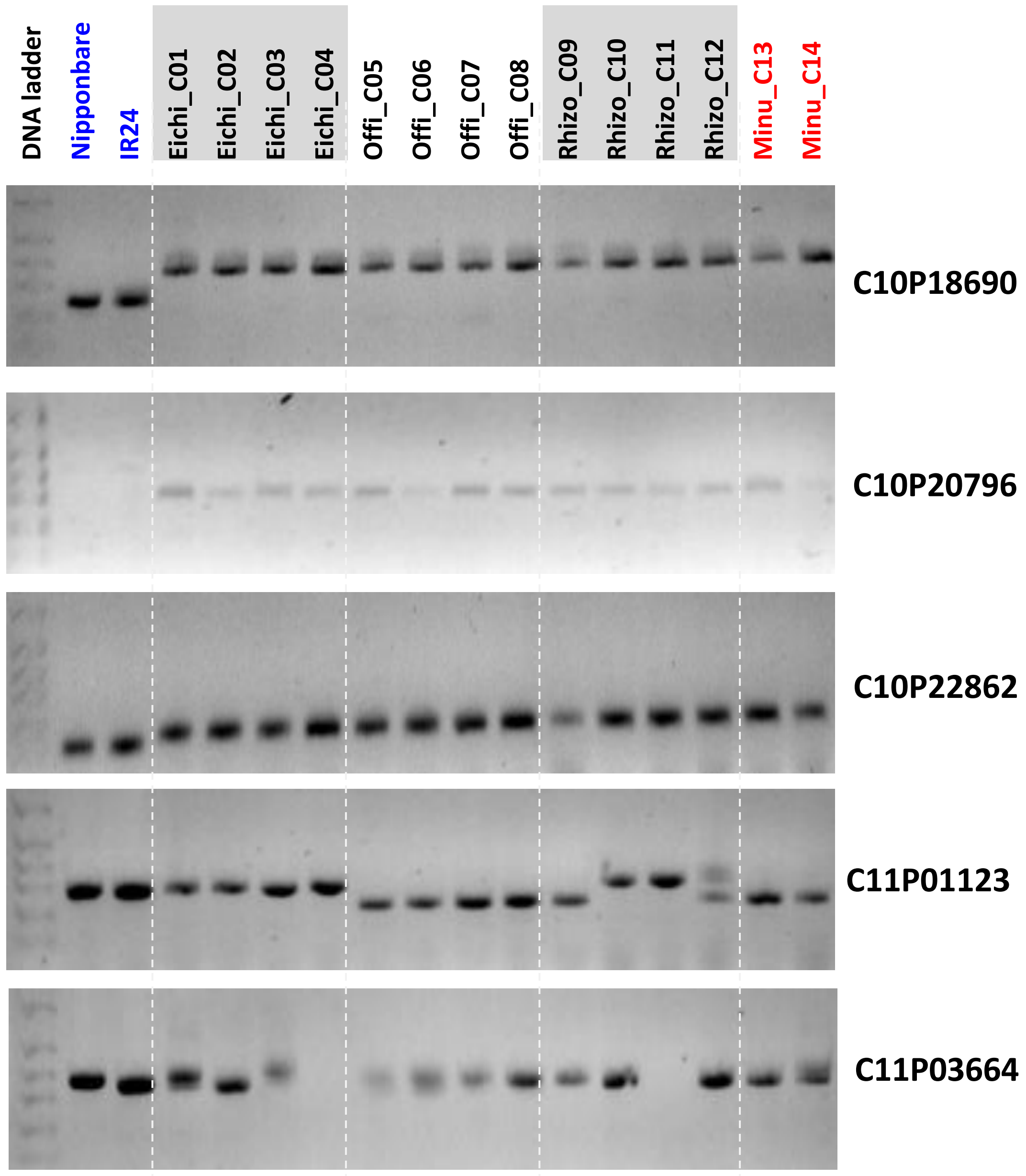
DNA ladder
Nipponbare
IR24
Eichi_C01
Eichi_C02
Eichi_C03
Eichi_C04
Offi_C05
Offi_C06
Offi_C07
Offi_C08
Rhizo_C09
Rhizo_C10
Rhizo_C11
Rhizo_C12
Minu_C13
Minu_C14
C10P18690
C10P20796
C10P22862
C11P01123
C11P03664

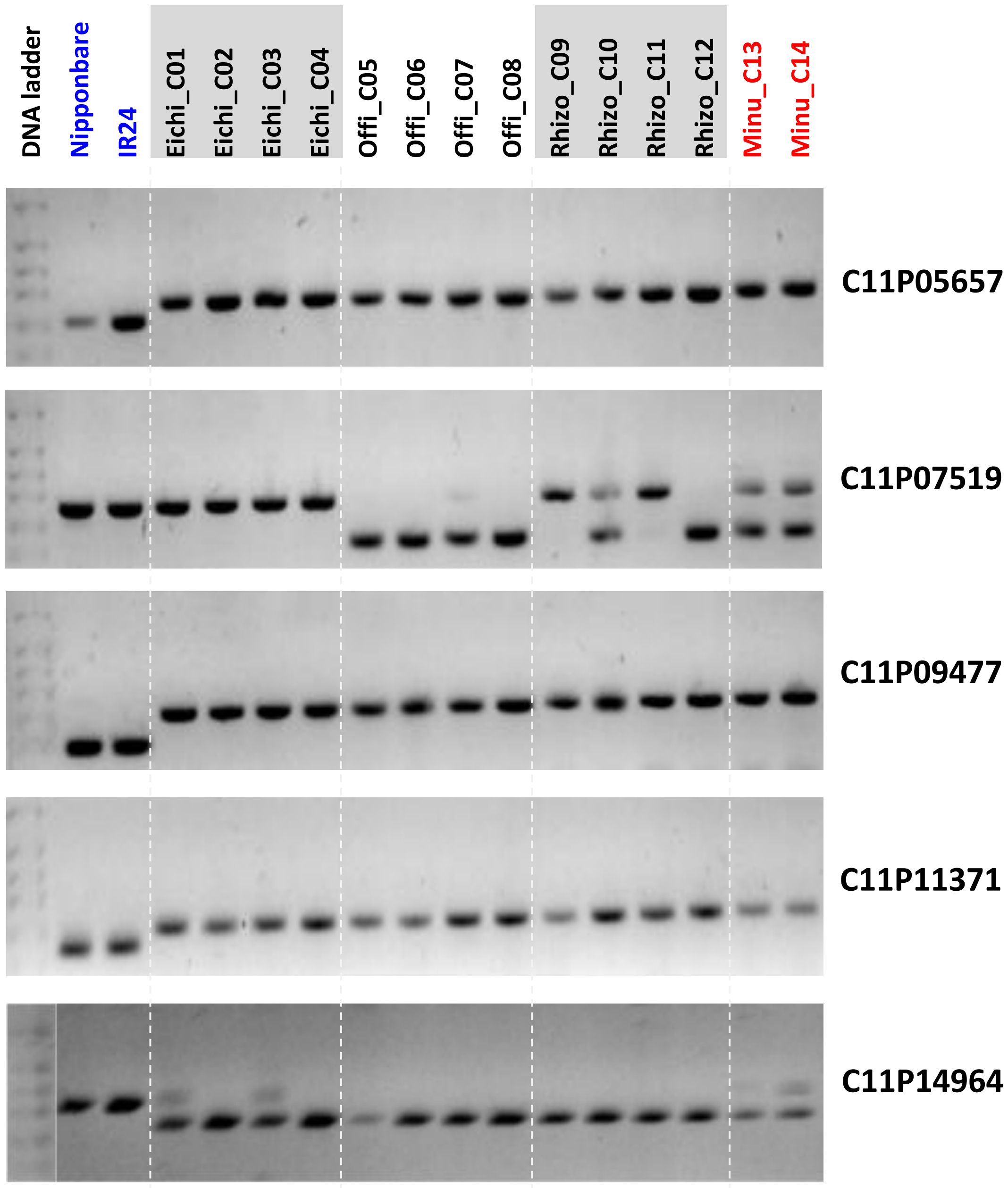
DNA ladder
Nipponbare
IR24
Eichi_C01
Eichi_C02
Eichi_C03
Eichi_C04
Offi_C05
Offi_C06
Offi_C07
Offi_C08
Rhizo_C09
Rhizo_C10
Rhizo_C11
Rhizo_C12
Minu_C13
Minu_C14
C11P05657
C11P07519
C11P09477
C11P11371
C11P14964

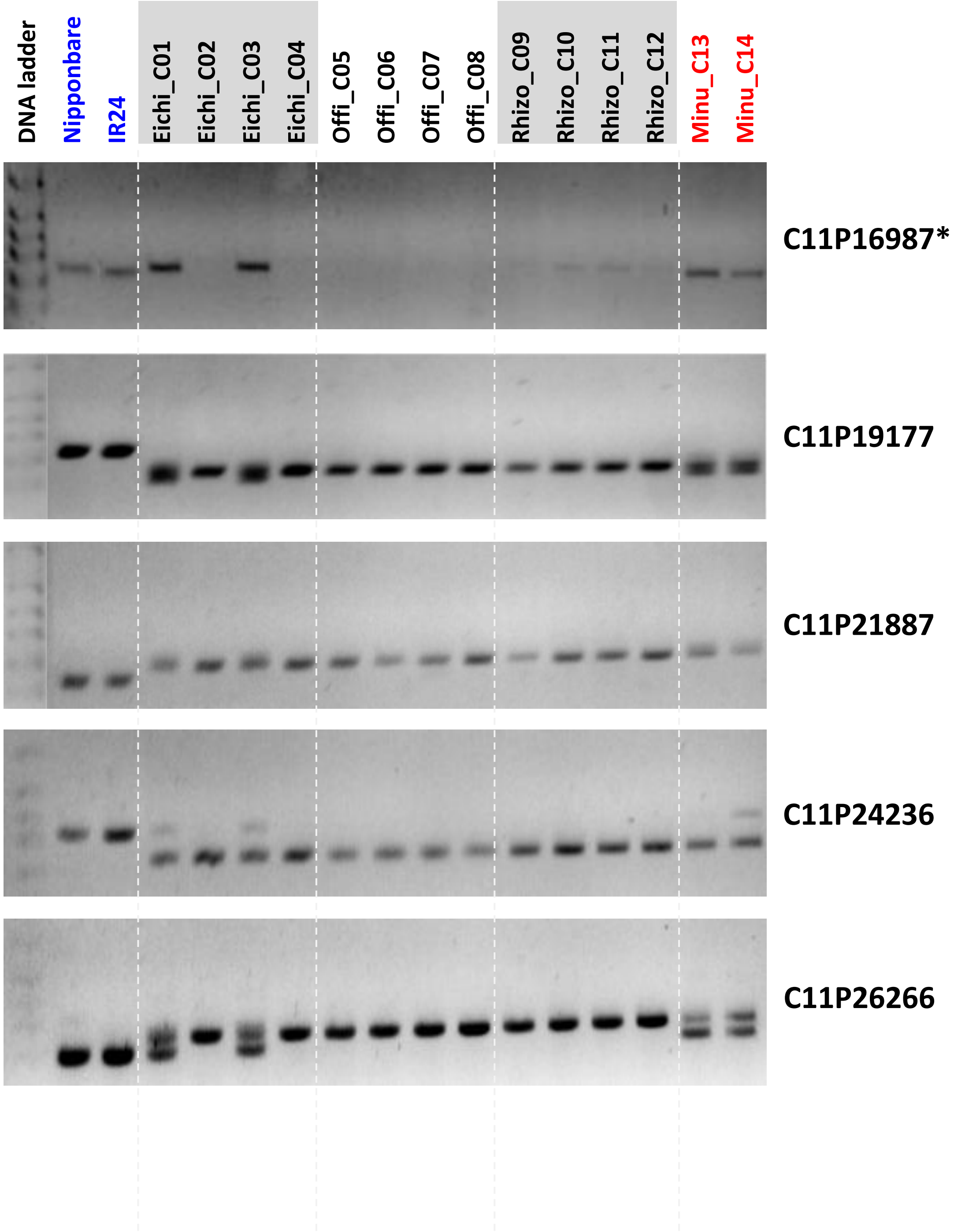
DNA ladder
Nipponbare
IR24
Eichi_C01
Eichi_C02
Eichi_C03
Eichi_C04
Offi_C05
Offi_C06
Offi_C07
Offi_C08
Rhizo_C09
Rhizo_C10
Rhizo_C11
Rhizo_C12
Minu_C13
Minu_C14
C11P16987*
C11P19177
C11P21887
C11P24236
C11P26266

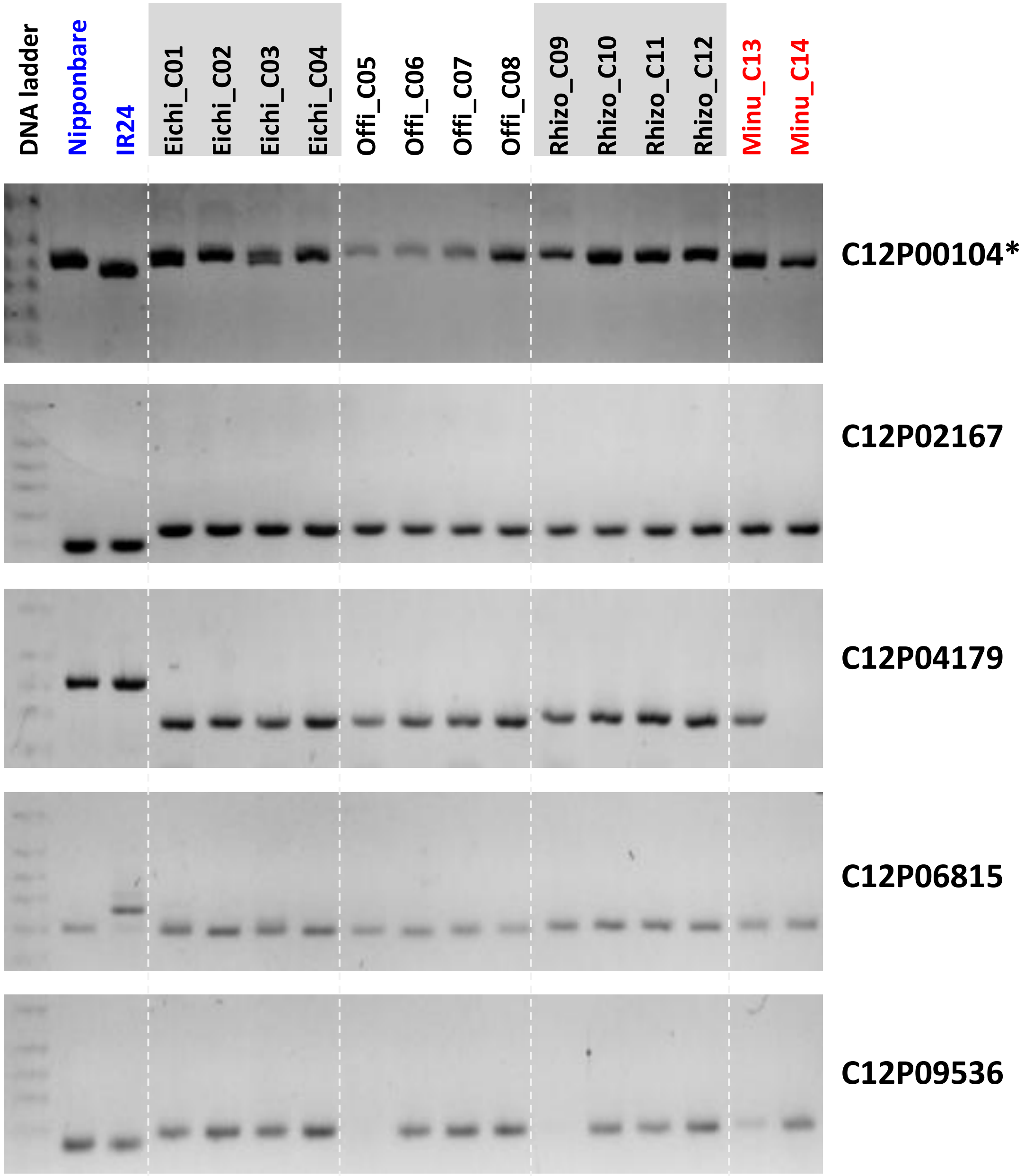
DNA ladder
Nipponbare
IR24
Eichi_C01
Eichi_C02
Eichi_C03
Eichi_C04
Offi_C05
Offi_C06
Offi_C07
Offi_C08
Rhizo_C09
Rhizo_C10
Rhizo_C11
Rhizo_C12
Minu_C13
Minu_C14
C12P00104*
C12P02167
C12P04179
C12P06815
C12P09536

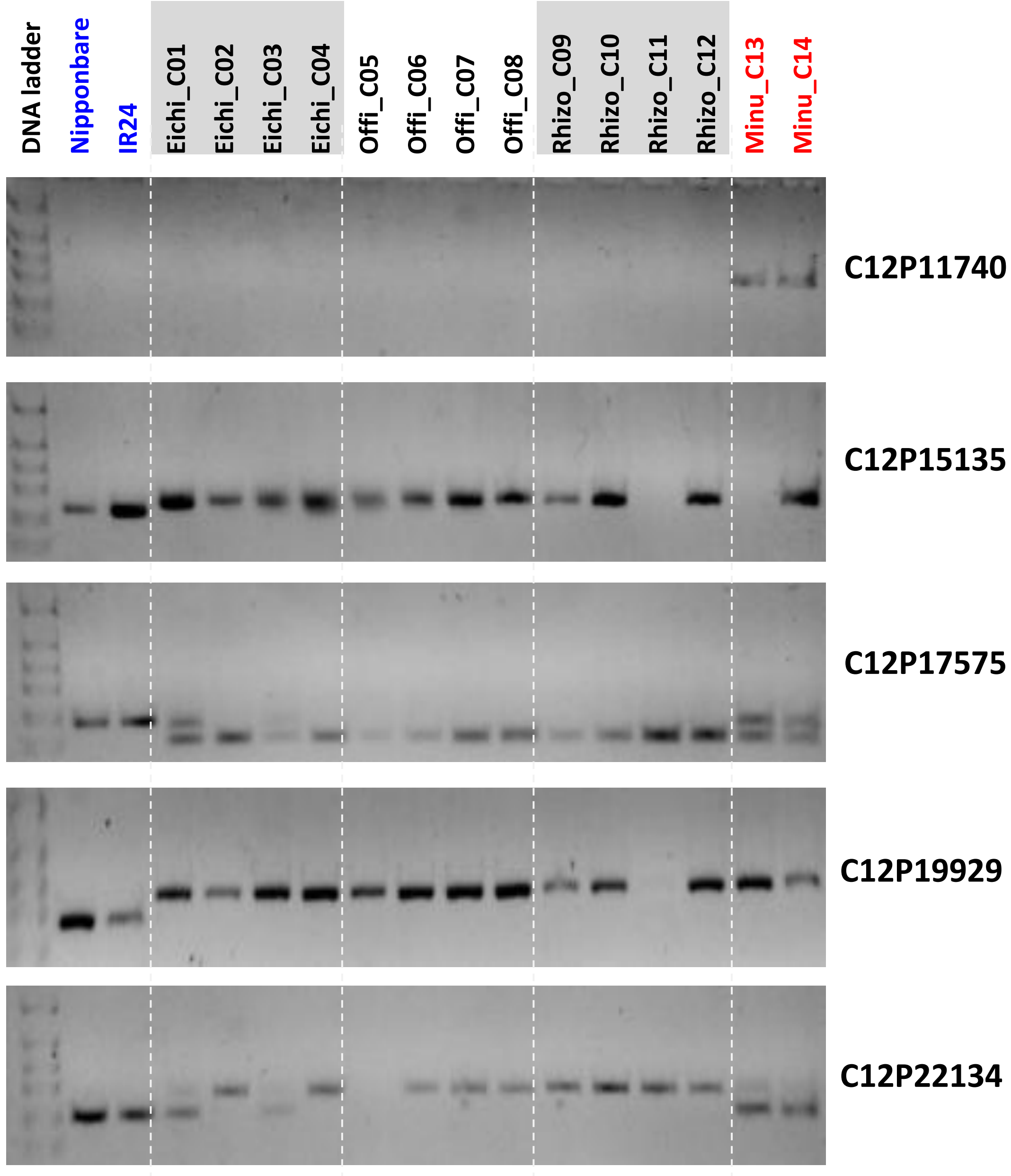
DNA ladder
Nipponbare
IR24
Eichi_C01
Eichi_C02
Eichi_C03
Eichi_C04
Offi_C05
Offi_C06
Offi_C07
Offi_C08
Rhizo_C09
Rhizo_C10
Rhizo_C11
Rhizo_C12
Minu_C13
Minu_C14
C12P11740
C12P15135
C12P17575
C12P19929
C12P22134

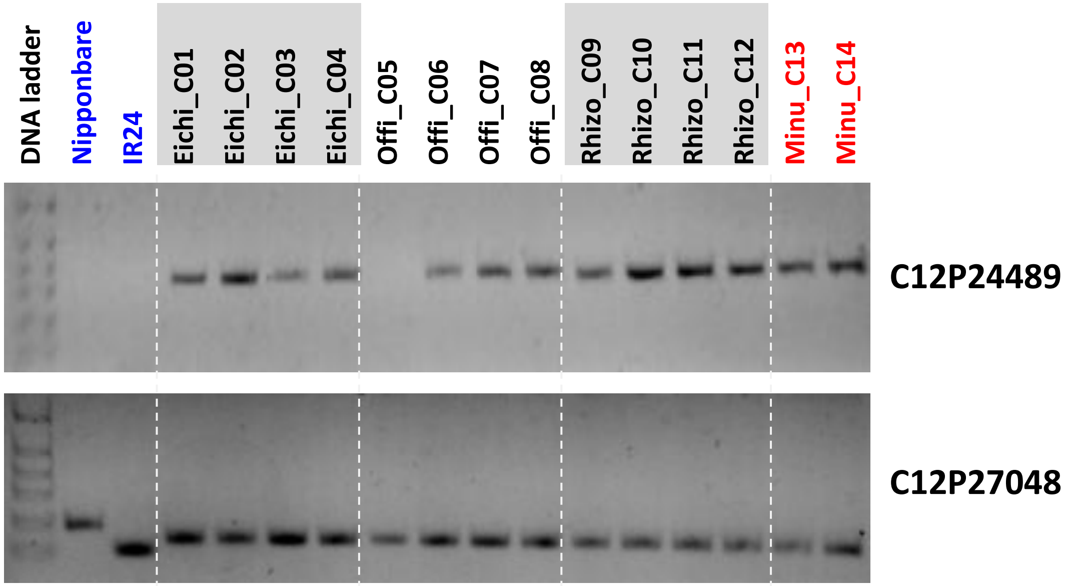
DNA ladder
Nipponbare
IR24
Eichi_C01
Eichi_C02
Eichi_C03
Eichi_C04
Offi_C05
Offi_C06
Offi_C07
Offi_C08
Rhizo_C09
Rhizo_C10
Rhizo_C11
Rhizo_C12
Minu_C13
Minu_C14
C12P24489
C12P27048
