# Supplementary material for: Development and validation of genome-wide polymorphic InDel marker set for harnessing the CC-genome wild rice species in the genus Oryza
Source: Front Plant Sci. 2026 Jan 28;17:1733586. doi: 10.3389/fpls.2026.1733586 (PMC12891111; doi:10.3389/fpls.2026.1733586)
Supplement: Supplementary file 3 [file DataSheet2.pdf]

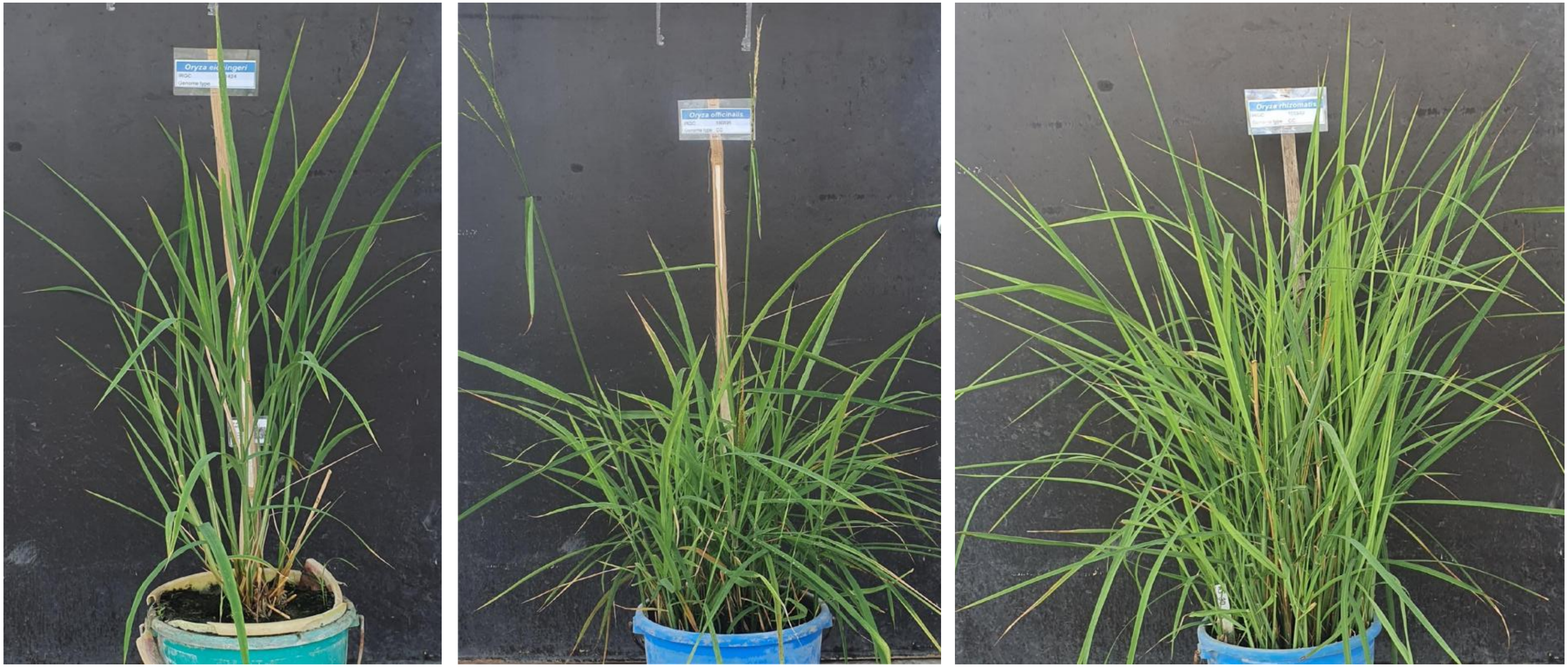

O. eichingeri
(IRGC 101424)

O. officinalis
(IRGC 100896)

O. rhizomatis
(IRGC 105949)

Supplementary Figure S2. Photos of the three CC-genome wild species (*O. eichingeri*, *O. officinalis*, and *O. rhizomatis*). Photos were taken at the wild rice glasshouse (No. AG02) at IRRI, Philippines.
